# Exploring the specialized metabolome of the plant pathogen *Streptomyces* sp. 11-1-2

**DOI:** 10.1038/s41598-024-60630-5

**Published:** 2024-05-06

**Authors:** Gustavo A. Díaz-Cruz, Dawn R. D. Bignell

**Affiliations:** 1 Department of Biology, Memorial University of Newfoundland, St. John’s, NL Canada; 2 Present Address: Phytopathology Department, Plant Protection Research Center (CIPROC), Agronomy School, Universidad de Costa Rica, San Jose, Costa Rica

**Keywords:** Microbiology, Bacteriology, Metabolomics

## Abstract

*Streptomyces* bacteria are notable for producing chemically diverse specialized metabolites that exhibit various bioactivities and mediate interactions with different organisms. *Streptomyces* sp. 11-1-2 is a plant pathogen that produces nigericin and geldanamycin, both of which display toxic effects against various plants. Here, the ‘One Strain Many Compounds’ approach was used to characterize the metabolic potential of *Streptomyces* sp. 11-1-2. Organic extracts were prepared from 11-1-2 cultures grown on six different agar media, and the extracts were tested in antimicrobial and plant bioassays and were subjected to untargeted metabolomics and molecular networking. Most extracts displayed strong bioactivity against Gram-positive bacteria and yeast, and they exhibited phytotoxic activity against potato tuber tissue and radish seedlings. Several known specialized metabolites, including musacin D, galbonolide B, guanidylfungin A, meridamycins and elaiophylin, were predicted to be present in the extracts along with closely related compounds with unknown structure and bioactivity. Targeted detection confirmed the presence of elaiophylin in the extracts, and bioassays using pure elaiophylin revealed that it enhances the phytotoxic effects of geldanamycin and nigericin on potato tuber tissue. Overall, this study reveals novel insights into the specialized metabolites that may mediate interactions between *Streptomyces* sp. 11-1-2 and other bacteria and eukaryotic organisms.

## Introduction

*Streptomyces* bacteria are ubiquitous in terrestrial and aquatic environments where they interact with various organisms in mutualistic or predatory/competitive ways. These interactions are thought to be primarily mediated by the production of specialized metabolites with diverse bioactivities^1^. The specialized metabolites can protect or inflict damage on a eukaryotic host or other microorganisms in the community in order to compete for nutritional resources, or they can act as signaling molecules in inter- and intra-species communication^2–7^.

In *Streptomyces*, the production of specialized metabolites is facilitated by biosynthetic gene clusters (BGCs) that can be categorized based on the core biosynthetic enzymes that are present and the class of metabolite(s) produced. For example, there are BGCs for the production of peptides, phenazines, polyketides, terpenes, lanthipeptides, non-ribosomal peptides, etc.^8–10^. Yet, not all specialized metabolites are produced at the same time or under the same environmental conditions, and this has led to the implementation of strategies such as the One Strain-Many Compounds (OSMAC) approach for exploring the metabolic diversity of microorganisms^11^. With this approach, growth conditions such as nutrient content, temperature and rate of aeration are altered in order to promote the activation of different specialized metabolite biosynthetic pathways and yield potentially novel compounds^11,12^. This strategy is complemented by easy access to genome sequencing and genome mining bioinformatics tools such as antiSMASH and the MIBiG database. Likewise, the use of untargeted metabolomics and the establishment of open specialized databases and cheminformatics tools like the Global Natural Products Social Molecular Networking (GNPS) platform and associated workflows has greatly facilitated and improved compound annotation^13–22^.

A small number of *Streptomyces* species can colonize and infect living plant tissues and cause different plant diseases, the most important of which is potato common scab (CS)^23^. This disease is primarily associated with the production of thaxtomin A, a phytotoxic specialized metabolite that functions as a cellulose biosynthesis inhibitor in plants^23^. More recently, other phytotoxic specialized metabolites such as borrelidin^24^ and desmethylmensacarcin^25^ have been proposed to participate in CS development by different *Streptomyces* species. In 2011, our lab isolated a highly pathogenic strain designated *Streptomyces* sp. 11-1-2 from a potato CS lesion, and it was later shown to produce two phytotoxic compounds, geldanamycin and nigericin, which may contribute to the pathogenic phenotype of the strain^26^. As the genome sequence of this strain is predicted to contain at least 53 BGCs for producing specialized metabolites^26^, it is possible that other compounds produced by this strain can also mediate plant-pathogen interactions as well as interactions with other microorganisms in the environment. The objective of this study was to employ the OSMAC principle together with genomic and metabolomic approaches and bioactivity analyses to characterize the specialized metabolome of *Streptomyces* sp. 11-1-2.

## Results and discussion

### 11-1-2 produces specialized metabolites with antimicrobial and phytotoxic activity

**Figure 1:**
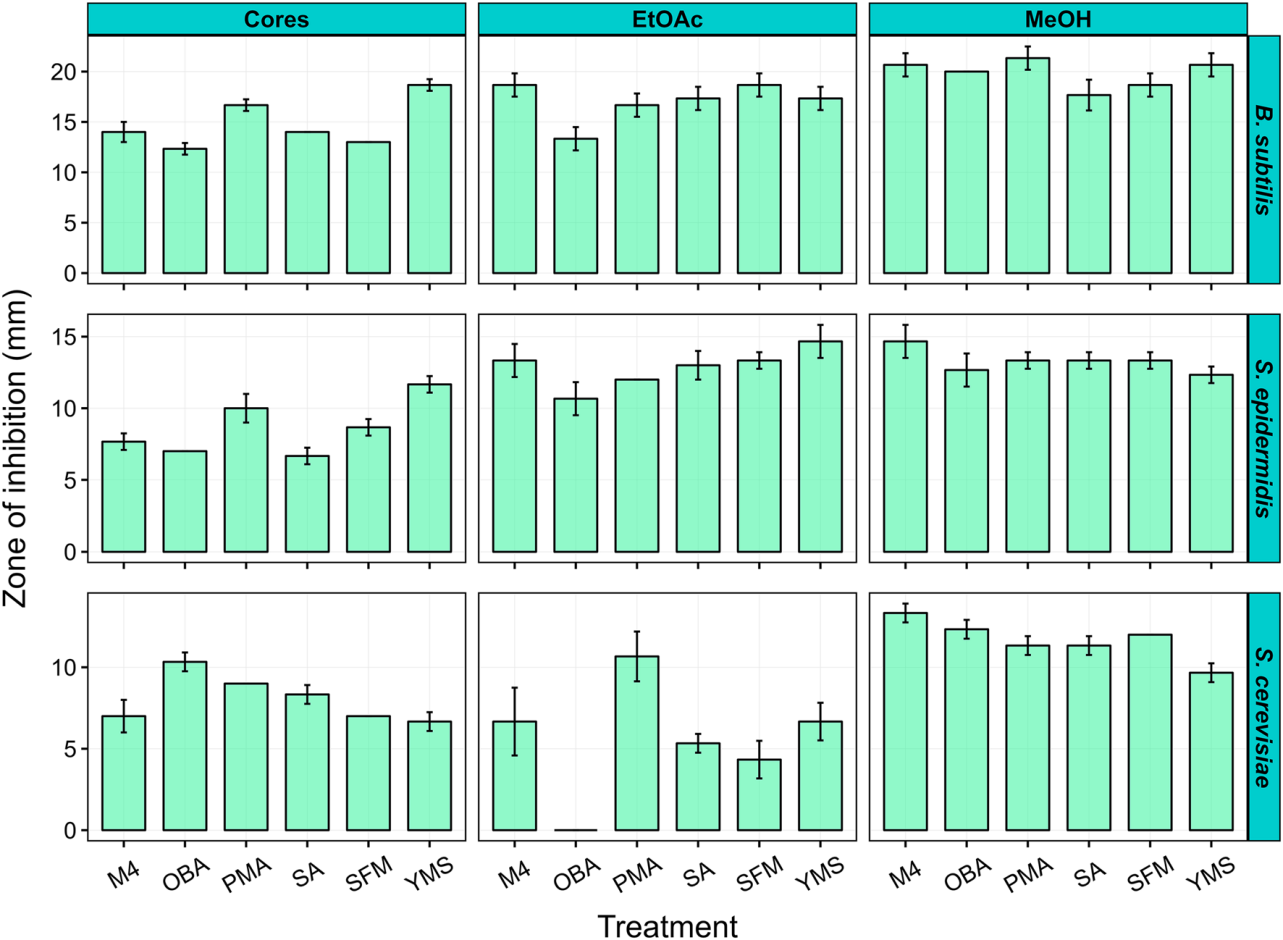
Antimicrobial assay using cores and organic culture extracts from *Streptomyces* sp. 11-1-2 plate cultures. Each bar represents the mean diameter of the zone of inhibition from duplicate samples for the cores and triplicate samples for the extracts (minus the diameter of the core or extract-containing paper disk), and error bars represent the standard deviation from the mean.

To gain new insights into the diversity of specialized metabolites produced by the 11-1-2 strain, we used the OSMAC approach to activate the production of as many compounds as possible. The strain was cultured on six different solid media, namely, M4 agar^27^, potato mash agar (PMA)^28^, oat bran agar (OBA)^29^, starch asparagine agar (SA)^30^, soy flour mannitol (SFM)^31^ agar and yeast extract-malt extract-starch (YMS) agar^32^. PMA was of special interest since it is derived from potato tuber flesh and is expected to at least partially mimic the nutritional conditions encountered during tuber colonization. Agar cores from the plate cultures were tested for phytotoxic activity against potato tuber tissue and for antimicrobial activity against different Gram-positive (*Bacillus subtilis*,* Staphylococcus epidermidis*) and Gram-negative [*Escherichia coli*,* Pseudomonas syringae* pathovar (pv.) *tomato*] bacteria and yeast (*Saccharomyces cerevisiae*). In addition, the plate cultures were subjected to extraction using two different organic solvents (ethyl acetate, EtOAc; methanol, MeOH), and the resulting extracts were evaluated for antimicrobial activity and for phytotoxic activity against both potato tuber tissue and radish seedlings. As shown in Fig. 1 and Supplementary Fig. S1 online, the agar cores and extracts all exhibited strong inhibitory activity against the two Gram-positive indicator organisms tested, whereas none showed activity against the Gram-negative indicator organisms (data not shown). The cores and extracts also exhibited antifungal activity against the yeast indicator organism except for the OBA EtOAc culture extract (Fig. 1 and Supplementary Fig. S1 online).

**Figure 2:**
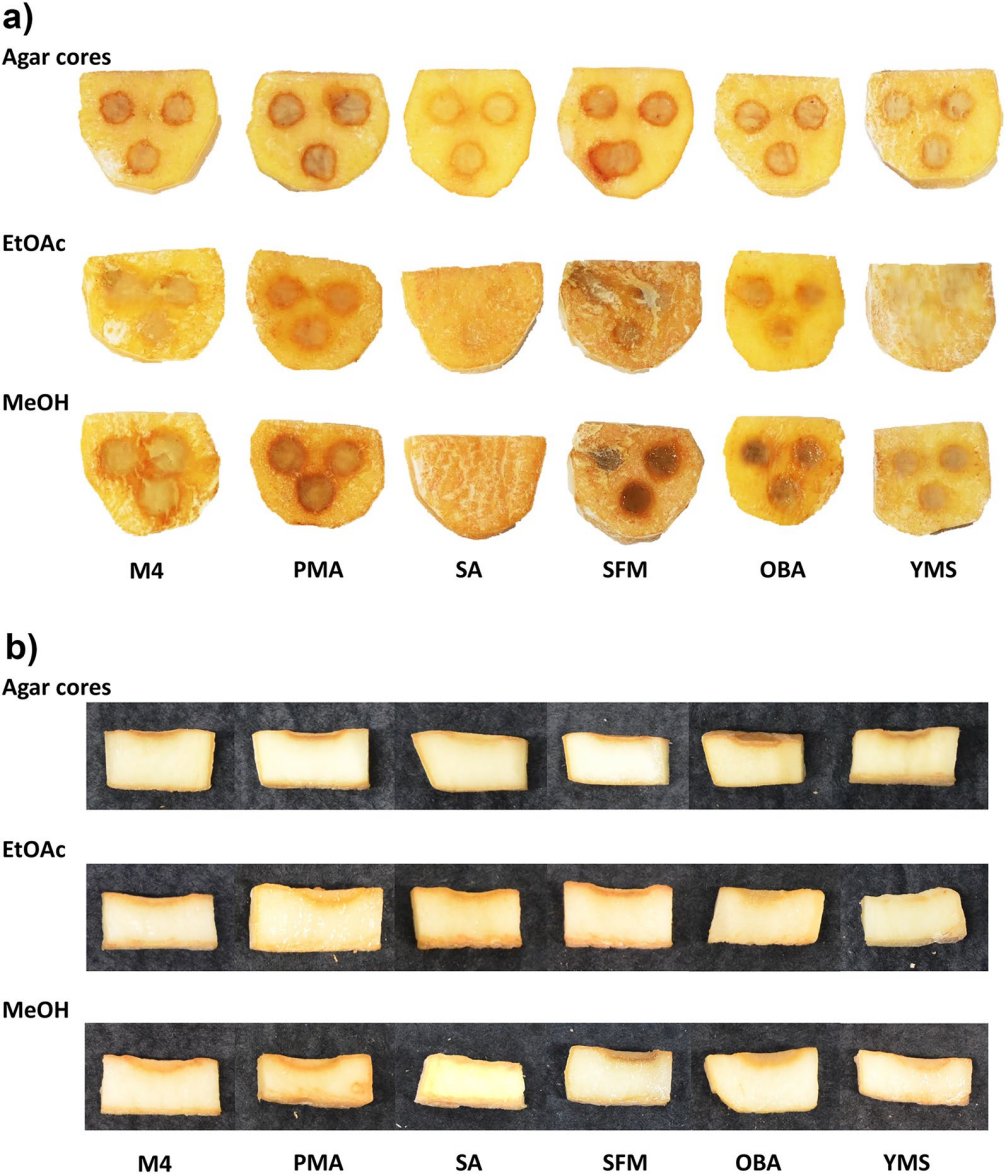
Potato tuber slice assay using cores and organic culture extracts from *Streptomyces* sp. 11-1-2 plate cultures. a) Top view and b) side view of a representative slice for each treatment.

Considering the role of 11-1-2 as a plant pathogen^33^, the production of phytotoxic compounds by this strain was of particular interest. Figure 2 shows that the agar cores from the different plate cultures exhibited varying levels of phytotoxicity against excised potato tuber tissue. Notably, the PMA, SFM and OBA cores were particularly phytotoxic and caused significant pitting and necrosis of the tuber tissue, whereas the SA cores had minimal effects on the tissue. The organic culture extracts exhibited similar effects as the corresponding agar cores, though in most cases the MeOH extracts were more active than the EtOAc extracts for a given plate culture. Overall, the PMA extracts were among the most phytotoxic for both solvents, while extracts from the SA cultures showed the least activity. The control cores and extracts from the uninoculated media had no effects on the tissue (Supplementary Fig. S2 online).

**Figure 3:**
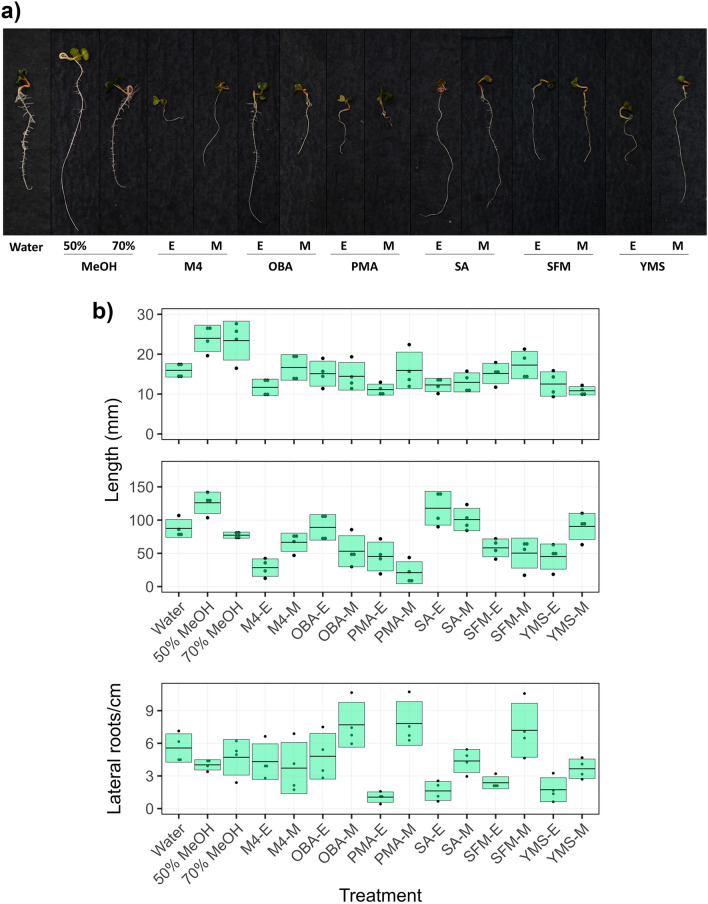
Effects of *Streptomyces* sp. 11-1-2 organic culture extracts on radish seedlings. a) Representative radish seedlings treated with different organic culture extracts. E: ethyl acetate; M: methanol. The photos were taken after five days of incubation. b) The shoot (top) and root (middle) length, and the number of lateral roots/cm of root length (bottom), were evaluated following treatment of germinated radish seeds with the organic culture extracts. Each box represents the mean measurement ± standard deviation. The name of the culture medium is followed by E (ethyl acetate) or M (methanol) according to the organic solvent used for the culture extraction. Methanol (50% v/v) served as the negative control for the methanol extracts, while methanol (70% v/v) served as the negative control for the ethyl acetate extracts.

When tested in a radish seedling bioassay, the culture extracts displayed some minor effects on shoot development, but the effects on seedling root growth were more pronounced (Fig. 3). In particular, the PMA MeOH and M4 EtOAc extracts both caused considerable stunting of the primary seedling root as compared to the other extracts. The SA extracts did not affect either root or shoot growth, but interestingly, the SA EtOAc extract did cause a noticeable decrease in lateral root development, whereas the M4 EtOAc extract had similar numbers of lateral roots/cm of root length as the control plants (Fig. 3). Inhibition of lateral root development was also observed with the PMA, SFM and YMS EtOAc extracts. The production of lateral roots is controlled by the presence of indole-3-acetic acid (IAA), which promotes cell division and maintains cell viability^34,35^. It is possible that IAA signaling interference by specialized metabolites within specific extracts such as the SA EtOAc is responsible for the observed effect on lateral root development. Auxin activity can be regulated or inhibited by a number of natural products^36^, some of which include cytokinins^37^, tryptophan conjugates of jasmonic acid and IAA^38^, and most notably, *Streptomyces*-produced terfestatin A^39^ and yokonolides A and B^40,41^.

### *Streptomyces* sp. 11-1-2 produces a diverse array of specialized metabolites

**Figure 4:**
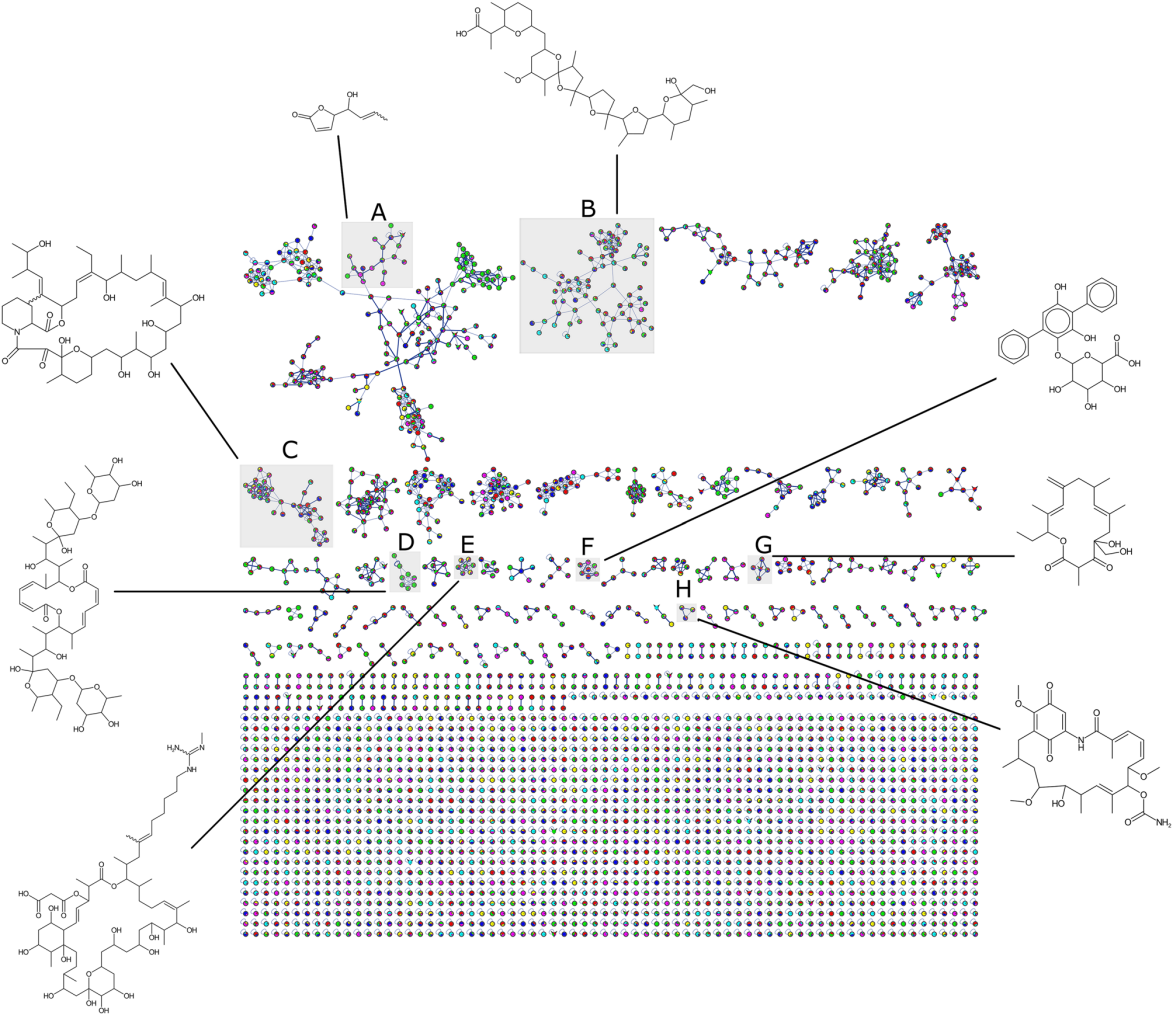
Ion identity molecular networks of *Streptomyces* sp. 11-1-2 metabolites. The metabolites were extracted from M4, OBA, PMA, SA, SFM and YMS plate cultures using ethyl acetate and were analyzed using untargeted LC-MS/MS in both positive and negative ionization mode. Each node in the networks represents one fragmentation spectrum, and nodes are linked if the cosine score is > 0.7 and there are at least 6 matched fragment ions. Networks containing selected specialized metabolites are highlighted and numbered based on the main metabolite: A, musacin D; B, nigericin; C, meridamycins; D, elaiophylin; E, guanidylfungin A; F, echoside C; G, galbonolide B; H, geldanamycin.

To further explore the specialized metabolites that are responsible for the observed bioactivities, the organic culture extracts were subjected to untargeted LC-MS/MS, and the resulting data were analyzed using ion identity molecular networking in the GNPS environment along with SIRIUS^42^ and BUDDY^43^ for molecular formula prediction and annotation of compounds. The molecular networking analysis revealed that the number of unique features was higher in the MeOH extracts than in the EtOAc extracts, and for both EtOAc and MeOH, the SA extracts had more unique features than the other media (Supplementary Fig. S3 online). The molecular networks for the EtOAc and MeOH culture extracts are shown in Fig. 4 and Supplementary Figure S4 online, respectively. The subnetworks for some predicted metabolites are numbered and highlighted.

Echosides are *para*-therphenyl compounds produced by *Streptomyces* spp., and some possess DNA topoisomerase inhibition activity and weak antibacterial activity^44^. In addition, echosides share structural similarities with terfestatin A, which has been shown to exhibit auxin signaling inhibitory activity^45^. The presence of three members of the echoside family was predicted in the EtOAc extracts from the different plate cultures, whereas none were found in the corresponding MeOH extracts (Supplementary Figs. S5−S7 online). Echoside C (**1**; *m*/*z* 453.1191, [M-H]^−^) was predicted in all the EtOAc media extracts, with the relative level being greatest in the PMA extract (Supplementary Fig. S5 online). However, the presence of this molecule could not be confirmed using targeted reverse-phase high performance liquid chromatography (RP-HPLC) (data not shown), suggesting that the amount produced in all the test media is very low. Echosides D and E (**2** and **3**; *m*/*z* 494.0914 [M-H]^−^ and *m/z* 524.1019 [M-H]^−^, respectively) were also predicted in the same network (Supplementary Figs. S5−S7 online). Other features in the echoside network include two matches to the GNPS library for the plant metabolites baicalin (**4**) and chrysin-7-O-glucoronide (**5**), and two more features for which the molecular formula were predicted by SIRIUS and BUDDY, but the structures remain unresolved. The production of plant metabolites, specifically flavonoids, by *Streptomyces* strains has been previously reported^46–48^, but the mechanism by which **4** and **5** are biosynthesized in 11-1-2 remains unclear.

Previous analysis of the 11-1-2 genome using antiSMASH version 6.0 revealed the presence of a region (#30) with 100% similarity to the reported BGC for echosides A-E^26^. This same region is now designated region 32 using the updated version of antiSMASH (version 7.1.0) (Supplementary Table S1 online). The echoside BGC was described as containing a NRPS-like polyporic acid synthase (encoded by *echA*) that is essential for biosynthesis of these metabolites, along with a number of genes with putative regulatory and tailoring functions^27^. BiG-SCAPE analysis showed that the echoside BGC and closely related BGCs can be subdivided into three gene cluster families (GCFs); moreover, the BGC network suggests that echosides and similar metabolites might be widespread among *Streptomyces* (Supplementary Fig. S5 online). Interestingly, a comparison between the BGC predicted at region 32 and the terfestatin/echoside BGC from *Streptomyces* sp. RM-5-8 showed important differences both upstream and downstream of the proposed core genes of the BGC (Supplementary Fig. S5 online). These differences might account for the production of different echoside analogues, as previously proposed^49^.

Elaiophylin is a macrolide with diverse bioactivities, including antimicrobial activity against some Gram-positive bacteria, and anticancer activity^50^. Elaiophylin is often co-produced with geldanamycin and nigericin, and several analogues like efomycins and halichoblelides^50–55^ have been identified from different *Streptomyces* strains of diverse origins. The production of elaiophylins and efomycins is likely performed by the same BGC, and some discrete differences may account for the production of the different analogues, as previously suggested^52^. The antiSMASH 7.1.0 analysis of the 11-1-2 genome revealed that region 46 has 100% similarity to the efomycins K/L BGC (Supplementary Table S1 online), which is also responsible for the production of elaiophylins^52^.

**Figure 5:**
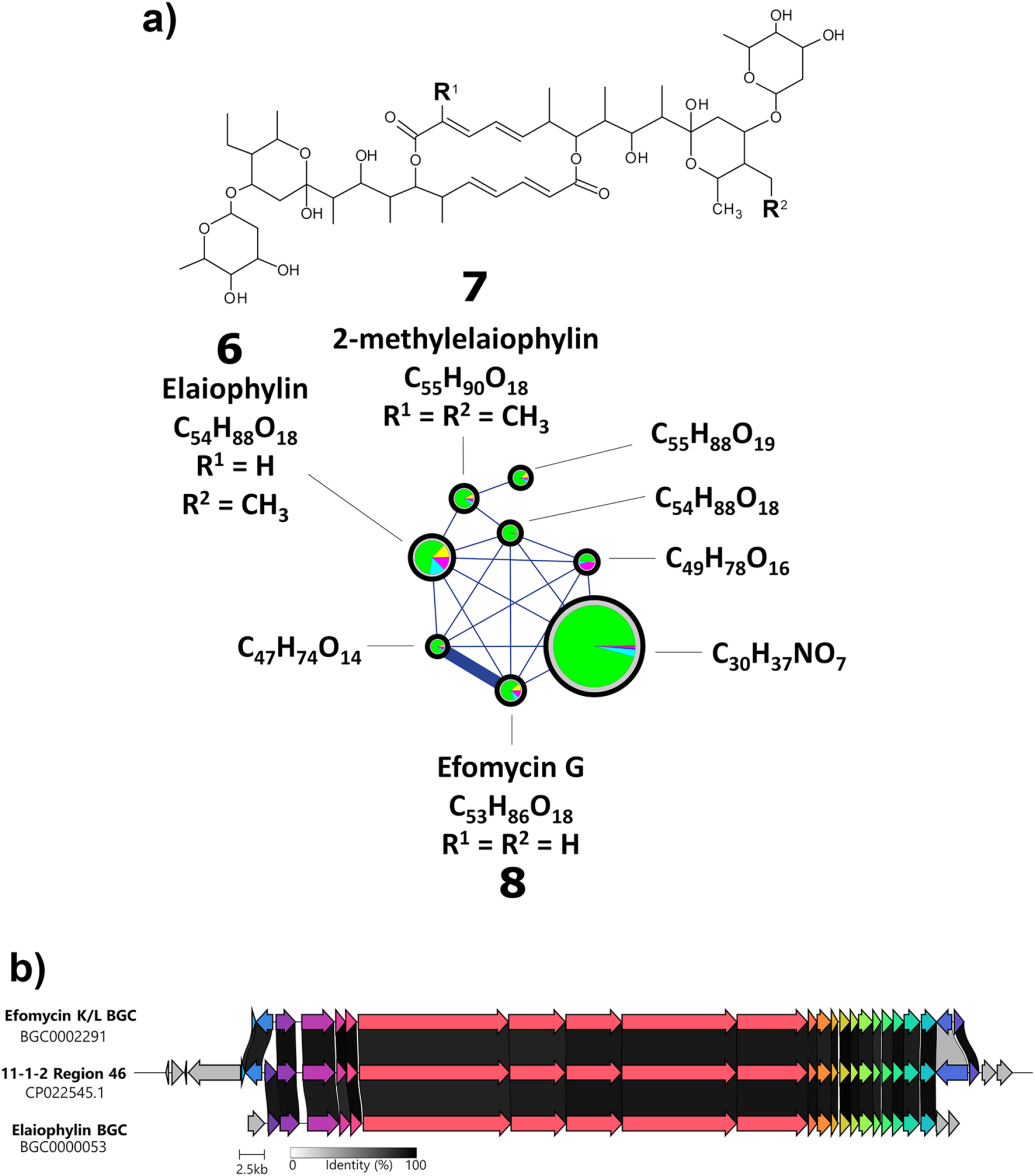
(**a**) Ion identity molecular network (negative ion mode) for elaiophylin metabolites in the *Streptomyces* sp. 11-1-2 culture extracts. Each node in the network represents one fragmentation spectrum, and the predicted molecular formula and structure of each is shown. Nodes are linked if the cosine score is > 0.7 and there are at least 6 matched fragment ions, and the thickness of the line increases at higher cosine values. The size of the node is relative to the total abundance of each compound in the analyzed culture extracts. The pie charts represent the relative abundance of each compound in the six different culture extracts: Red = M4, Yellow = OBA, Green = PMA, Teal = SA, Blue = SFM, Violet = YMS. (**b**) Alignment of the elaiophylin BGC and the efomycin K/L BGC available in the MIBiG database and the predicted elaiophylin BGC at region 46 in *Streptomyces* sp. 11-1-2. Genes coloured the same belong to the same functional group, and homologues are linked by shaded areas that indicate the % amino acid identity of the corresponding protein products.

The untargeted LC-MS/MS analysis revealed the presence of a feature in negative ion mode that is consistent with elaiophylin (**6**; *m*/*z* 1023.5900, [M-H]^−^ and 1069.5951, [M+FA]^−^) (Fig. 5 and Supplementary Figs. S8 and S9 online). Targeted detection by RP-HPLC (Supplementary Fig. S10 online) confirmed the presence of this compound in all the extracts except for the M4 and SFM extracts, and the levels were significantly higher in the PMA extracts than in the other extracts, with the PMA EtOAc extract having the greatest levels (Table 1 and Fig. 5). A number of related features were also detected, most notably, 2-methylelaiophylin (**7**; *m*/*z* 1037.6052, [M-H]^−^ and 1083.6106, [M+FA]^−^), and efomycin G (**8**; *m*/*z* 1009.5739, [M-H]^−^ and 1055.5799, [M+FA]^−^) (Supplementary Figs. S8 and S9 online), which possess antimicrobial activity against Gram-positive bacteria^50,56^. Intriguingly, other compounds predicted in these networks do not match the known elaiophylin analogues, suggesting the possible presence of novel elaiophylin derivatives being produced by 11-1-2.

**Table 1:** Nigericin, geldanamycin and elaiophylin quantification in different organic culture extracts.

Extraction solvent	Culture medium	Nigericin (mM)	Geldanamycin (mM)	Elaiophylin (mM)
Ethyl acetate	M4	6.93 ± 0.55	0.19 ± 0.02	N.D.^2^
	OBA	0.82 ± 0.28	0.22 ± 0.02	0.02 ± 0.01
	PMA	9.44 ± 2.26	0.51 ± 0.04	1.21 ± 0.11
	SA	1.58 ± 0.17	0.01 ± 0.00	0.02 ± 0.01
	SFM	4.53 ± 0.37	0.15 ± 0.02	N.D.^2^
	YMS	8.48 ± 0.66	0.15 ± 0.01	0.02 ± 0.00
Methanol	M4	2.93 ± 0.50	0.03 ± 0.04	N.D.^2^
	OBA	0.98 ± 0.43	0.15 ± 0.09	0.03 ± 0.01
	PMA	3.79 ± 2.32	0.18 ± 0.04	0.27 ± 0.04
	SA	1.00 ± 0.23	N.D.^2^	0.01 ± 0.00
	SFM	1.42 ± 0.33	0.05 ± 0.02	N.D.^2^
	YMS	4.10 ± 0.89	0.06 ± 0.01	0.01 ± 0.00

^1^The values correspond to the average of three replicates ± one standard deviation.

^2^Not detected.

**Figure 6:**
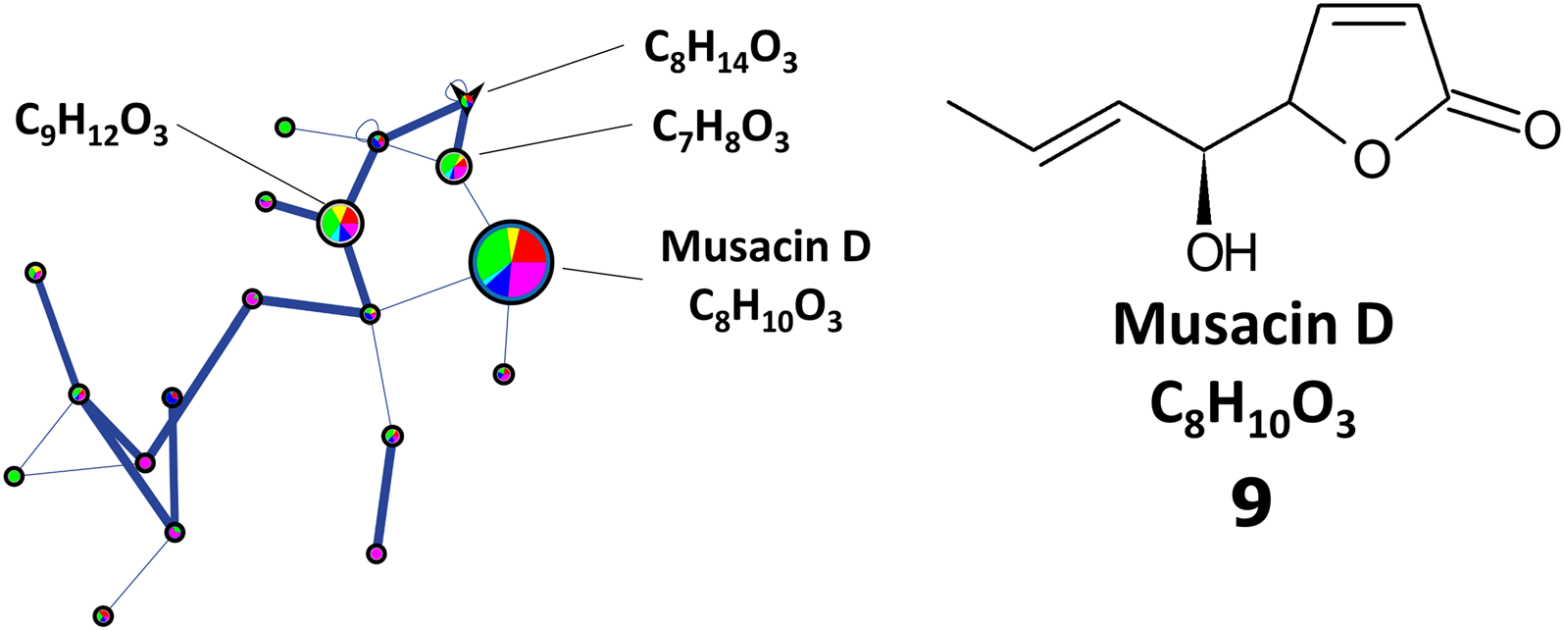
Ion identity molecular network (positive ion mode) for musacin D in the *Streptomyces* sp. 11-1-2 culture extracts. The features of the network are as described in the legend for Fig. 5.

A prominent feature found in the LC-MS/MS dataset was predicted to be musacin D (**9**,* m*/*z* 155.0706 [M+H]^+^), and the relative level of this feature was greatest in the PMA and YMS EtOAc (Fig. 6 and Supplementary Fig. S11 online). Musacin D is among several musacins first detected in *Streptomyces griseoviridis*, and all were initially reported as having no significant bioactivity^57,58^. Later, it was found that the plant pathogen *Nigrospora sacchari* produces a phytotoxic compound with an identical structure to musacin D except for a different stereochemical configuration of the hydroxy group at the carbon five position^59^. With the current information, it is not possible to conclude if the feature predicted as musacin D has a role in the phytotoxicity of the extracts, or if its identity truly corresponds to musacin D or the phytotoxin from *N. sacchari*. There is also currently no information regarding the genes necessary for the biosynthesis of musacins, and thus it was not possible to determine if 11-1-2 contains the genes necessary to produce these compounds. Purification and characterization of the feature will be necessary to establish its significance for the pathogenic phenotype of 11-1-2.

Niphimycins are macrolides with structural resemblance to azalomycins and guanidylfungins, all of which have antifungal activity^60–63^. The analysis of the different extracts identified a niphimycin-like compound with a match to the GNPS library in the majority of the extracts (**10**; *m*/*z* 1130.7292, [M+H]^+^ and 1128.7141, [M-H]^−^) (Supplementary Figs. S12−S14 online). This compound is likely to be guanidylfungin A, a molecule with antimicrobial activity against fungi and Gram-positive bacteria and found to be co-produced with guanidylfungin B^64^. Guanidylfungin B was also predicted to be present in some of the extracts (**11**; *m*/*z* 1116.7121, [M+H]^+^) (Supplementary Figs. S12−S14 online). The presence of guanidylfungin A in the extracts may account for some of the observed antifungal activity against the *S. cerevisiae* indicator strain (Fig. 1). However, as this compound was detected in the OBA EtOAc extract that had no observable antifungal activity in our bioassays, further investigation of this compound and its production by 11-1-2 is required. To date, there is no BGC assigned specifically for the production of guanidylfungins in *Streptomyces*. Given the structural similarities between niphimycins and guanidylfungins, the cluster in region 50 (87% similarity to the niphimycins C-E cluster; Supplementary Table S1 online) of the 11-1-2 genome is most likely responsible for production of this compound (Supplementary Fig. S12 online).

A feature predicted to be galbonolide B (**12**) was also detected in the extracts (*m*/*z* 365.2315, [M+H]^+^) (Supplementary Figs. S15 and S16 online). Galbonolide B is a 14-membered macrolide with antifungal activity^65^. Region 5 in the 11-1-2 genome is predicted by antiSMASH 7.1.0 to contain a BGC with 20% similarity to the known rustmicin BGC (Supplementary Table S1 online). Rustmicin is also known as galbonolide A, and the related compound neorustmicin is also known as galbonolide B; both molecules are 14-membered macrolides with antifungal activity^65^. The rustmicin BGC from *Streptomyces galbus* contains 30 genes and is separated into two operons. Genes *galABCDE* are sufficient for the production of neorustmicin/galbonolide B^66^, while genes *galGHIJK* are required for production of rustmicin/galbonolide A^65^. The role of the remaining genes is unclear, but it is speculated that they are tailoring enzymes^65,67^. The *galABCDE* core is present in region 5 of 11-1-2, suggesting that this strain can at least produce galbonolide B, while the flanking genes could serve as post-polyketide synthase tailoring enzymes yielding novel galbonolides (Supplementary Fig. S15 online).

Meridamycin is a 27-membered macrolide that was first isolated from a strain of *Streptomyces hygroscopicus*^68^*.* Meridamycin binds to the human FKBP12 protein more efficiently than the closely related specialized metabolites FK506 or rapamycin, thus inhibiting the target of rapamycin (TOR) complex involved in immune responses^68^. The FKBP12 protein is also present in plants, where it participates in different development pathways associated with the TOR complex^69,70^. The use of rapamycin shows limited effects on seedling development^71,72^, but the use of ATP-competitive TOR inhibitors reduces Arabidopsis root growth^72^. It is not clear how meridamycin would affect plant development, but its higher binding efficiency compared to rapamycin makes it a metabolite of interest in the context of the plant pathogen 11-1-2. Two features predicted to be meridamycin (**13**; *m*/*z* 820.5215, [M-H]^−^ and 822.5331, [M+H]^+^) and its analogue meridamycin A (**14**; *m*/*z* 838.5325, [M-H]^−^ and 862.5268, [M+Na]^+^) (Supplementary Figs. S17−S19 online) were detected in the extracts, and region 11 of the 11-1-2 genome partially matches the reported meridamycin BGC (Supplementary Table S1 online). Other meridamycins analogues (meridamycin B, C, D) have been described in *Streptomyces* sp. LZ35 and are likely to be produced by the same BGC as meridamycin^73^. The meridamycin BGC in *Streptomyces* sp. NRRL 30748 and *Streptomyces* sp. DSM 4137 (syn: *Streptomyces malaysiensis* DSM 4137) is classified as a hybrid non ribosomal peptide-polyketide synthase (NRP-PKS) cluster^74,75^, and the 11-1-2 cluster shows some differences compared to these reported clusters (Supplementary Fig. S17 online). Firstly, the 11-1-2 region and the meridamycin BGC show two PKS genes, *merC* and *merD*, while the cluster sequenced in *Streptomyces* sp. DSM 4137 contains a single PKS. Secondly, a group of four genes encoding two putative transcriptional regulators (LysR- and TetR-family), one transporter (major facilitator superfamily, MFS) and a DNA polymerase III subunit alpha is located upstream of the PKS genes in 11-1-2, whereas they are situated downstream of the PKS genes in *S. malaysiensis* and are absent from the *Streptomyces* sp. NRRL 30748 cluster. Thirdly, three genes located downstream from the PKS genes in 11-1-2 are not present in the other clusters. Two of the genes encode a predicted two-component sensor histidine kinase and a LuxR transcriptional regulator, while the third gene encodes a protein of unknown function. It remains unclear how these genomic differences might affect the production of meridamycins in 11-1-2.

Previously, we reported that 11-1-2 produces geldanamycin and nigericin along with putative intermediates and other predicted related compounds^26^. Molecular networks for these compounds were again identified in the current study, though the geldanamycin network had fewer features compared to our previous analysis^26^. Notably, a feature from the nigericin network (**15;** *m*/*z* 784.5188, [M+NH_4_]^+^) was predicted in the EtOAc and MeOH extracts from all media, but it was more prominent in both the PMA and SA organic culture extracts (Supplementary Fig. S20 online). This feature was not previously detected in the cultures of 11-1-2, and it is unclear if this compound exhibits similar biological activity as nigericin. Targeted analysis revealed that the nigericin production levels were significantly greater than that of geldanamycin in all the media tested (Table 1). Both compounds were detected in both the EtOAc and MeOH extracts, with the EtOAc extracts containing higher amounts of each. Of the media tested, PMA supported the highest amounts of both compounds, while SA contained the least amount of each (Table 1).

### Elaiophylin enhances the phytotoxic effects of geldanamycin and nigericin on potato tuber tissue

Nigericin and geldanamycin can inhibit the radicle growth of various crop and weed species of plants, and in a mixture they cause necrosis and pitting of excised potato tuber tissue^26^. Intriguingly, they are often co-produced with elaiophylin, which is structurally similar to the known phytotoxic compound concanamycin A^23,76^. As there is currently no information on the phytotoxic activity of elaiophylin, we sought to investigate whether this compound can exhibit phytotoxic effects against plant tissues on its own and whether it influences the activity of nigericin and/or geldanamycin.

**Figure 7:**
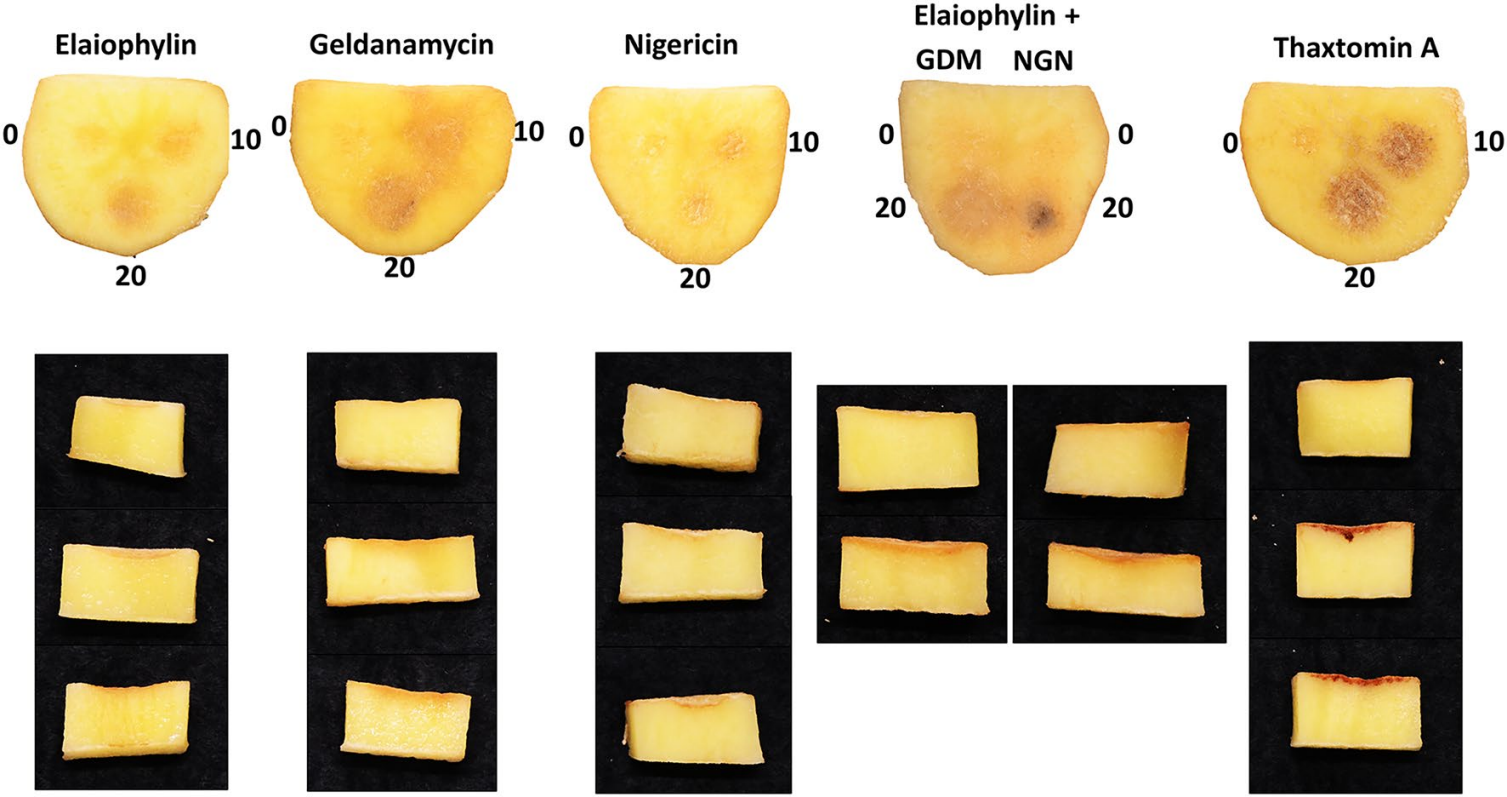
Top and side view of potato tuber slices treated with pure compounds. Each tuber slice contained disks inoculated with 0 (control), 10, and 20 nmol of the respective compound in a fixed volume of 20 μL. For the combination of elaiophylin with geldanamycin or nigericin, each compound provided half of the amount reported, i.e., 20 nmol had 10 nmol of each compound. The assay was performed twice with three biological replicates per treatment in each assay, with similar results obtained each time.

Figure 7 shows the results of a potato tuber slice bioassay conducted using different amounts of pure nigericin, geldanamycin, and elaiophylin. On its own, elaiophylin (10 nmol) caused some minor necrosis and pitting to the tissue, and the damage was greater when a higher amount (20 nmol) of the compound was administered. The combination of elaiophylin with geldanamycin or nigericin increased the severity of tissue damage caused by each compound independently, especially nigericin. As with geldanamycin and nigericin, the effects of elaiophylin were distinct from the damage caused by the thaxtomin A phytotoxin at the same concentrations.

The bioactivity of elaiophylin was also tested against radish seedlings alongside geldanamycin, nigericin and thaxtomin A (Supplementary Fig. S21 online). The shoot length was slightly increased in the presence of elaiophylin as compared to the control plants, while the other compounds seemed to reduce shoot length, although these changes were not statistically significant (Supplementary Fig. S21 online, top panel). In contrast, the root length was reduced in presence of higher concentrations of elaiophylin when compared to the DMSO-treated control plant, though the results were not statistically significant (Supplementary Fig. S21 online, bottom panel). The combination of elaiophylin with geldanamycin or nigericin did not affect the root length when compared to the same concentrations of each compound separately. As previously observed (Supplementary Fig. S21 online, bottom panel), thaxtomin A exhibited the greatest effect on root growth in the bioassay.

As some of the 11-1-2 culture extracts inhibited radish seedling lateral root development (Fig. 3), this variable was also evaluated following treatment with the pure compounds. The addition of elaiophylin did not have a negative impact on this variable, but it slightly improved the number of roots when combined with geldanamycin (Supplementary Fig. S22 online). Interestingly, the use of nigericin alone or in combination caused a clear reduction in the length and number of lateral roots/cm of root length (Supplementary Figs. S22 and S23 online), an effect not previously described for this compound. This suggests that the observed reduction in lateral root development by the 11-1-2 culture extracts may be due in part to the presence of nigericin in the extracts. However, given that low numbers of lateral roots/cm were observed with some organic extracts containing lower amounts of nigericin (e.g., SA-E), while some extracts containing higher nigericin levels (e.g. M4-E) did not have an effect on lateral root development, it is likely that other compounds in the extracts are also contributing to the observed effects on lateral root development, an idea that warrants further investigation.

We also investigated the bioactivity of pure echoside C against plants since this compound was predicted in the EtOAc culture extracts (Supplementary Figs. S5−S7 online), and it is related to the auxin signaling inhibitor terfestatin A. However, the compound did not exhibit any significant effects on excised potato tuber tissue or on radish seedlings in our bioassays (Supplementary Figs. S24−S26 online), suggesting that it likely does not contribute to the phytotoxic activity of the 11-1-2 strain.

## Conclusions

In this study, we showed that the plant pathogen *Streptomyces* sp. 11-1-2 is capable of producing various types of specialized metabolites with bioactivity against Gram-positive bacteria, yeast and plants. Growth of the strain on the PMA medium generated the highest yields of the phytotoxic compounds geldanamycin and nigericin, and agar cores and extracts from this medium were very bioactive in all the assays performed. Considering that 11-1-2 was originally isolated from a CS-diseased potato tuber, it is likely that the strain has adapted its metabolism to enable colonization of the nutrient-dense tuber tissues, thereby providing a selective advantage over other microbes in the soil environment.

The untargeted LC-MS/MS and molecular networking predicted the presence of many known specialized metabolites, including musacin D, galbonolide B, guanidylfungin A, meridamycins and elaiophylin, as well as closely related compounds with unknown structure and bioactivity. Along with geldanamycin and nigericin, elaiophylin was found to be produced in greatest amounts in PMA, and we showed for the first time that pure elaiophylin enhances the tuber tissue damage caused by these phytotoxins, suggesting that the co-production of nigericin, geldanamycin and elaiophylin might contribute to the plant pathogenic phenotype of 11-1-2. The elaiophylin precursors pteridic acids A and B, and related pteridic acids H and F exhibit protective effects against abiotic stress and auxin-like effects^77,78^. However, these compounds were not detected in our study, and so it is unclear whether they contribute to host interactions with the 11-1-2 strain. Elaiophylin has been reported to display good antibacterial activity against Gram-positive bacteria along with efomycin G, which was also predicted to be present in the organic extracts^55^. Thus, it is possible that elaiophylin and related molecules might also contribute to interactions between the 11-1-2 strain and other Gram-positive bacteria in the environment. Additionally, the production of meridamycins and their mode of action make them an attractive group of metabolites for further investigation, as they might be involved in interfering with plant signaling and development^70^.

An interesting result from this study was the observation that pure nigericin has a negative impact on lateral root development, an effect not previously recorded for this compound. The mechanism for how this occurs remains unclear and warrants further investigation. It is notable that the SA EtOAc extract contained low nigericin amounts and yet caused a considerable reduction in lateral root development of the radish seedings, whereas the M4 EtOAc extract had significantly higher levels of nigericin but was not as effective in reducing lateral root development. Furthermore, the MeOH extracts were generally more phytotoxic against the potato tuber tissue than the EtOAc extracts, even though the EtOAc extracts contained equivalent or higher levels of nigericin, geldanamycin and elaiophylin. This suggests that other compounds may be contributing to the phytotoxic effects of the 11-1-2 strain. Some prominent compounds in the culture extracts could not be identified in this study, and a few BGCs predicted in the genome have no known products, which could represent novel compounds. Whether any of these contribute to the plant pathogenic phenotype of the 11-1-2 strain will require further investigation.

## Materials and methods

### Bacterial strains, culture conditions and general procedures

*Streptomyces* sp. 11-1-2 was originally isolated from a potato common scab lesion in Newfoundland, Canada^33^. The strain was routinely cultured on potato mash agar (PMA)^28^ at 28 °C and was maintained at − 80 °C as a glycerol spore stock^31^. For analysis of specialized metabolite production, 30 µL of a spore stock was spread onto four plates of M4^27^, oat bran agar (OBA)^29^, PMA, starch asparagine agar (SA)^30^, soy flour mannitol (SFM)^31^ and yeast extract-malt extract-starch (YMS)^32^ solid media, and the plates were incubated at 28 °C for 14 days. *Escherichia coli* DH5α (New England Biolabs Canada) was routinely cultured on Luria Bertani (LB) Miller agar (Fisher Scientific, USA) at 37 °C, and *Staphylococcus epidermidis* ATCC 14990 (ATCC) was routinely cultured on tryptic soy agar (TSA) (BD Biosciences, Canada) at 37 °C. *Bacillus subtilis* ATCC 23857 (ATCC) and *Pseudomonas syringae* pv. *tomato* DC3000^79^ were grown on TSA at 28 °C, while *Saccharomyces cerevisiae* ATCC 208352 (ATCC) was grown on yeast extract-peptone-dextrose (YPD) agar at 28 °C. For long-term storage of *E. coli*,* S. epidermidis*, *B. subtilis*,* P. syringae* and *S. cerevisiae*, 20% v/v glycerol stocks were prepared and kept at − 80 °C^80^.

### Organic extraction of *Streptomyces* sp. 11-1-2 cultures

Culture extracts were prepared from the 11-1-2 plate cultures as follows. The agar in each plate was cut in half, and each half was further cut into small pieces using a sterile pipettor tip. The pieces were transferred to two clean 50 mL plastic conical tube and the agar pieces were stored at − 80 °C until ready for extraction. The agar pieces were first thawed at room temperature before addition of the organic solvent. ACS grade EtOAc or MeOH (20 mL) was added to each vessel, and the contents were mixed and left to incubate at room temperature overnight. Following incubation, the extracts were filtered using Whatman® #1 filter paper (GE Healthcare Life Sciences) and transferred into clean conical tubes. The remaining agar pieces were rinsed with 10 mL of fresh solvent, which was subsequently filtered and combined with the corresponding extract. The solvent was removed from each extract by rotary evaporation using an IKA® RV 10 instrument (IKA Works, USA). Methanol extracts were also subjected to freeze-drying for 3 days using a Labconco Freezone 12 Freeze Dryer (Labconco Corp., MO, USA) to remove the remaining aqueous liquid following rotary evaporation. The dried MeOH extracts were resuspended in 1 mL of 50% LC-grade MeOH, while the dried EtOAc extracts were redissolved in 1 mL of 70% v/v LC-grade MeOH. The extracts were then stored at − 80 °C. Non-inoculated plates of each medium were used as controls in all cases. The control plates were prepared, incubated, and subjected to organic extraction alongside the inoculated media.

### LC-MS/MS analysis

Untargeted metabolomics analysis of the culture extracts was conducted at the BioZone Mass Spectrometry Facility in the University of Toronto using a Thermo Scientific Ultimate 3000 UHPLC coupled to Thermo Scientific Q-Exactive equipped with a HESI-II probe. Metabolite separation was carried out using a Thermo Scientific Hypersil Gold C18 column (2.1 × 50 mm, 1.9 µm, 175 Å, equipped with guard column) maintained at 40 °C and a mobile phase gradient of water/acetonitrile with 0.1% v/v formic acid. Mass spectra were recorded using fast polarity switching. Then, the raw LC-MS/MS data files were converted into mzXML format and separated based on their polarity using MSConvert for further analysis. Both the raw and the converted files are available in the Mass Spectrometry Interactive Virtual Environment (MassIVE) data repository (massive.ucsd.edu) under the accession number MSV000091858.

### Molecular networking

The raw data files were pre-processed using MSConvert^81^ with the recommended parameters (https://ccms-ucsd.github.io/GNPSDocumentation/fileconversion/). The files were converted into mzXML files with a 32-bit binary encoding precision and no zlib compression. The Peak Picking filter was selected with the vendor algorithm for MS-Levels 1-2.

The spectral data obtained were analyzed using Feature-Based Molecular Networking (FBMN)^20,21^ complemented with Ion Identity Molecular Networking^22^. For this, the mzXML files were imported and analyzed using MZmine (version 2.37.corr17.7)^82^. The parameters used for the analysis are detailed in Supplementary Table S2 online. The peak area of each ion in the feature quantification table was adjusted by subtracting the area from the corresponding control extract. The spectral summary files (.mgf files), edited feature quantification tables (.csv files) and supplementary edge files (.csv files) were then processed using the FBMN workflow^21^ within the GNPS web platform (https://gnps.ucsd.edu). The parameters of the FBMN analysis are detailed in Supplementary Table S3 online. The networks generated were visualized using Cytoscape^83^. To further characterize the results in each network, the spectra of compounds without matches to the GNPS reference libraries were analyzed using MetFrag^84^, SIRIUS, including the CSI:fingerID option^42,85^ and BUDDY with no chemical database restriction^43^.

### Targeted detection of *Streptomyces* metabolites

Detection of geldanamycin in culture extracts was performed by RP-HPLC using an Agilent 1260 Infinity Quaternary LC system (Agilent Technologies Canada Inc., Mississauga, ON) as described previously^26^. A standard curve was generated using known amounts of a pure geldanamycin standard (Cayman Chemicals, USA) and was used for metabolite quantification.

Nigericin was detected using a modified version of a previously published protocol^26^. Organic culture extracts were analyzed using an Agilent 1260 Infinity LC-6230 TOF LC-MS system. Extracts (5 µL) from triplicate cultures were loaded onto a Zorbax SB C-18 column (4.6 × 150 mm, 5 µm particle size; Agilent Technologies Canada Inc.) held at 22 °C. The column was equilibrated in 20% ammonium acetate buffer (20 mM):80% MeOH, and compounds were eluted using a linear gradient to 100% MeOH over 22.5 min at a constant flow rate of 1 mL/min. Data acquisition was performed using Agilent MassHunter version B.08.00 (Agilent Technologies Canada Inc.) and MestReNova version 14.1.2 (Mestrelab Research S.L.) was used for data analyses. Quantification of nigericin was achieved by generating a standard curve using known amounts of a pure nigericin sodium salt standard (Cayman Chemicals, USA).

Detection of elaiophylin in culture extracts was performed by RP-HPLC using an Agilent 1260 Infinity Quaternary LC system. Extracts (5 µL) prepared from triplicate cultures were loaded onto a Poroshell 120 EC-C18 column (4.6 × 50 mm, 2.7 µm particle size; Agilent Technologies Canada Inc.) held at 40 °C. The initial mobile phase consisted of 70% H_2_O:30% MeOH, and this was held constant for 0.5 min followed by a linear gradient to 100% MeOH over 15 min. The mobile phase was maintained at this concentration for 1 min, and was then returned to 70% H_2_O:30% MeOH over 1 min. The flow rate was held constant at 1 ml/min. Elaiophylin was monitored using a detection wavelength of 254 nm, and the ChemStation software version B.04.03 (Agilent Technologies Canada Inc.) was used for data acquisition. A standard curve was generated using known amounts of a pure elaiophylin standard (Cayman Chemicals, USA) and was used for metabolite quantification.

Detection of echoside C in culture extracts was performed by RP-HPLC using an Agilent 1260 Infinity Quaternary LC system. Extracts (5 µL) prepared from triplicate cultures were loaded onto a Poroshell 120 EC-C18 column (4.6 × 50 mm, 2.7 µm particle size) held at 40 °C. Metabolites were eluted using a linear gradient of acetonitrile (ACN) and H_2_O with 0.1% v/v formic acid in both phases. The initial mobile phase consisted of 95% H_2_O:5% ACN, and this was held constant for 0.5 min before changing to 5% H_2_O:95% ACN over a period of 10 min. The mobile phase was maintained at this concentration for 1 min, and was then returned to 95% H_2_O:5% ACN over 1 min. The flow rate was held constant at 1 mL/min. Echoside C was monitored using a detection wavelength of 260 nm, and the ChemStation software version B.04.03 was used for data acquisition. A known amount of a pure echoside C standard (Chemspace LLC, USA) was used as a reference.

### Plant bioassays

The phytotoxic activity of agar cores, organic culture extracts and pure compounds was evaluated using a potato tuber slice bioassay as described before^26,86^. The potato tubers used in the assays were purchased from local vendors. The phytotoxicity of extracts and pure compounds was also evaluated using a radish seedling assay as previously described^26^. Following incubation, the root and shoot length of each seedling was measured, and the number of lateral roots per centimeter of total root length was determined using a dissecting microscope^34^. The radish seeds (cv Cherry Belle) used in the assays were purchased from McKenzie Seeds Canada. All experiments/protocols involving plant materials were performed with relevant institutional, national, and international guidelines and legislation.

### Antimicrobial bioassays

The antimicrobial activity of 11-1-2 agar cores and culture extracts were determined using different microbial indicator organisms. Cultures of *B. subtilis*, *S*. *epidermidis* and *P. syringae* pv. *tomato* DC3000 were prepared by inoculating a single colony into 10 mL of TSB, while *S. cerevisiae* cultures were prepared by inoculating a single colony into 10 mL of YPD broth. The cultures were incubated overnight at 28 °C (*B*. *subtilis*, *P*. *syringae* pv. *tomato*, *S*. *cerevisiae*) and 37 °C (*S. epidermidis*), and then 2 mL of each was added to 200 mL of molten TSA (for bacterial cultures) or YPD agar (for yeast cultures). The cells were mixed with the melted agar by swirling, and the agar was then poured into sterile Corning® Square BioAssay dishes (245 mm × 245 mm) and allowed to solidify. Agar cores from 11-1-2 cultures plates, or 6 mm Whatman® filter disks (GE Healthcare Life Sciences) with 20 µL of 11-1-2 culture extract, were placed equidistantly onto the bioassay plates, after which the plated were incubated at the appropriate temperature for 24 h. The diameter of the zone of inhibition around the agar core or paper disk was recorded after the incubation period.

### Bioinformatics analyses

To identify specialized metabolite biosynthetic gene clusters in 11-1-2, the genome sequence (NCBI accession NZ_CP022545) was analyzed using antiSMASH 7.1.0^87^ with all the extra features available. The 11-1-2 genome was also analyzed and compared using the BiG-SCAPE workflow against the MIBiG database with the default parameters (https://git.wur.nl/medema-group/BiG-SCAPE)^88^. For the large-scale network and phylogenetic analysis of specific BGCs, 2136 Streptomycetales genomes (as of November 2022) were downloaded from the National Center for Biotechnology Information (NCBI) and were processed using the command-line version of antiSMASH 5.1.2 with the bacterial setting and otherwise default parameters. Sequence similarity networks and phylogenetic relationships for the BGCs were generated using the BiG-SCAPE workflow with the default parameters (https://git.wur.nl/medema-group/BiG-SCAPE)^88^. Network files were visualized using Cytoscape version 3.8.2^83^, and the BGCs present within each network were retrieved and compared using Clinker with default parameters and Clustermap.js. was used to visualize the BGC alignment results^89^. Further visualization of selected BGCs was performed using the CAGECAT suite^90^ and Gene Graphics^91^.

### Statistical analyses

The results of the radish seedling bioassays (root and shoot length) using the pure compounds were analyzed using an analysis of variance and Tukey’s test using the R package “agricolae”, and visualized using “ggplot2”, “ggsignif” and “patchwork”^92–95^.

### Supplementary Information

Supplementary Information.

## Data Availability

The metabolomics dataset generated and analyzed during the current study is available in the Mass Spectrometry Interactive Virtual Environment (MassIVE) data repository (massive.ucsd.edu) under the accession number MSV000091858.

## References

[1] O’Brien J, Wright GD (2011). An ecological perspective of microbial secondary metabolism. Curr. Opin. Biotechnol..

[2] Scott JJ (2008). Bacterial protection of beetle-fungus mutualism. Science.

[3] Oh DC, Scott JJ, Currie CR, Clardy J (2009). Mycangimycin, a polyene peroxide from a mutualist *Streptomyces* sp.. Org. Lett..

[4] Kim DR (2019). A mutualistic interaction between *Streptomyces* bacteria, strawberry plants and pollinating bees. Nat. Commun..

[5] Traxler MF, Kolter R (2015). Natural products in soil microbe interactions and evolution. Nat. Prod. Rep..

[6] Cornforth DM, Foster KR (2013). Competition sensing: The social side of bacterial stress responses. Nat. Rev. Microbiol..

[7] Vaz Jauri P, Kinkel LL (2014). Nutrient overlap, genetic relatedness and spatial origin influence interaction-mediated shifts in inhibitory phenotype among *Streptomyces* spp.. FEMS Microbiol. Ecol..

[8] Lee N (2020). Mini review: Genome mining approaches for the identification of secondary metabolite biosynthetic gene clusters in *Streptomyces*. Comput. Struct. Biotechnol. J..

[9] Nett M, Ikeda H, Moore BS (2009). Genomic basis for natural product biosynthetic diversity in the actinomycetes. Nat. Prod. Rep..

[10] Hwang KS, Kim HU, Charusanti P, Palsson BT, Lee SY (2014). Systems biology and biotechnology of *Streptomyces* species for the production of secondary metabolites. Biotechnol. Adv..

[11] Bode HB, Bethe B, Höfs R, Zeeck A (2002). Big effects from small changes: Possible ways to explore nature’s chemical diversity. ChemBioChem.

[12] Romano S, Jackson SA, Patry S, Dobson ADW (2018). Extending the “one strain many compounds” (OSMAC) principle to marine microorganisms. Mar. Drugs.

[13] Terlouw, B. R. *et al.* MIBiG 3.0: A community-driven effort to annotate experimentally validated biosynthetic gene clusters. *Nucleic Acids Res.* **51**, epub ahead of print (2022). 10.1093/nar/gkac1049 PMC9825592 36399496

[14] Blin K (2021). AntiSMASH 6.0: Improving cluster detection and comparison capabilities. Nucleic Acids Res..

[15] Bayona LM, de Voogd NJ, Choi YH (2022). Metabolomics on the study of marine organisms. Metabolomics.

[16] van Bergeijk DA, Terlouw BR, Medema MH, van Wezel GP (2020). Ecology and genomics of Actinobacteria: New concepts for natural product discovery. Nat. Rev. Microbiol..

[17] Wu C, Kim HK, Van Wezel GP, Choi YH (2015). Metabolomics in the natural products field—A gateway to novel antibiotics. Drug Discov. Today Technol..

[18] Kind T (2018). Identification of small molecules using accurate mass MS/MS search. Mass Spectrom. Rev..

[19] Chaleckis R, Meister I, Zhang P, Wheelock CE (2019). Challenges, progress and promises of metabolite annotation for LC-MS-based metabolomics. Curr. Opin. Biotechnol..

[20] Wang M (2016). Sharing and community curation of mass spectrometry data with global natural products social molecular networking. Nat. Biotechnol..

[21] Nothias LF (2020). Feature-based molecular networking in the GNPS analysis environment. Nat. Methods.

[22] Schmid R (2021). Ion identity molecular networking for mass spectrometry-based metabolomics in the GNPS environment. Nat. Commun..

[23] Li Y, Liu J, Díaz-Cruz G, Cheng Z, Bignell DRD (2019). Virulence mechanisms of plant-pathogenic *Streptomyces* species: An updated review. Microbiology.

[24] Cao Z, Khodakaramian G, Arakawa K, Kinashi H (2012). Isolation of Borrelidin as a phytotoxic compound from a potato pathogenic *Streptomyces* strain. Biosci. Biotechnol. Biochem..

[25] Lapaz, M. I. *et al.* Isolation and structural characterization of a non-diketopiperazine phytotoxin from a potato pathogenic *Streptomyces* strain. *Nat. Prod. Res.* **0**, 1–7 (2018). 10.1080/14786419.2018.1511554 30304960

[26] Díaz-Cruz GA, Liu J, Tahlan K, Bignell DRD (2022). Nigericin and geldanamycin are phytotoxic specialized metabolites produced by the plant pathogen *Streptomyces* sp. 11-1-2. Microbiol. Spectr..

[27] Zhu J (2014). Identification and catalytic characterization of a nonribosomal peptide synthetase-like (NRPS-like) enzyme involved in the biosynthesis of echosides from *Streptomyces* sp. LZ35. Gene.

[28] Fyans JK, Altowairish MS, Li Y, Bignell DRD (2015). Characterization of the coronatine-like phytotoxins produced by the common scab pathogen *Streptomyces scabies*. Mol. Plant Microbe Interact..

[29] Johnson EG, Joshi MV, Gibson DM, Loria R (2007). Cello-oligosaccharides released from host plants induce pathogenicity in scab-causing *Streptomyces* species. Physiol. Mol. Plant Pathol..

[30] Paradkar AS, Jensen SE (1995). Functional analysis of the gene encoding the clavaminate synthase 2 isoenzyme involved in clavulanic acid biosynthesis in *Streptomyces clavuligerus*. J. Bacteriol..

[31] Kieser T, Bibb MJ, Buttner MJ, Chater KF, Hopwood DA (2000). Practical Streptomyces Genetics.

[32] Ikeda H, Kotaki H, Tanaka H, Omura S (1988). Involvement of glucose catabolism in avermectin production by *Streptomyces avermitilis*. Antimicrob. Agents Chemother..

[33] Fyans JK, Bown L, Bignell DRD (2016). Isolation and characterization of plant pathogenic *Streptomyces* species associated with common scab-infected potato tubers in Newfoundland. Phytopathology.

[34] Celenza JL, Grisafi PL, Fink GR (1995). A pathway for lateral root formation in *Arabidopsis thaliana*. Genes Dev..

[35] Du Y, Scheres B (2018). Lateral root formation and the multiple roles of auxin. J. Exp. Bot..

[36] Hayashi KI (2021). Chemical biology in auxin research. Cold Spring Harb. Perspect. Biol..

[37] Stoeckle D, Thellmann M, Vermeer JE (2018). Breakout—lateral root emergence in *Arabidopsis thaliana*. Curr. Opin. Plant Biol..

[38] Staswick PE (2009). The tryptophan conjugates of jasmonic and indole-3-acetic acids are endogenous auxin inhibitors. Plant Physiol..

[39] Yamazoe A, Hayashi KI, Kepinski S, Leyser O, Nozaki H (2005). Characterization of terfestatin A, a new specific inhibitor for auxin signaling. Plant Physiol..

[40] Hayashi K-I, Ogino K, Oono Y, Uchimiya H, Nozaki H, Yokonolide A (2001). A new inhibitor of auxin signal transduction, from *Streptomyces diastatochromogenes* B59. J. Antibiot..

[41] Hayashi KI, Yokonolide B (2003). A novel inhibitor of auxin action, blocks degradation of AUX/IAA factors. J. Biol. Chem..

[42] Dührkop, K. *et al. SIRIUS 4: A Rapid Tool for Turning Tandem Mass Spectra into Metabolite Structure Information*. *Nature Methods* vol. 16 (Springer, 2019). 10.1038/s41592-019-0344-8 30886413

[43] Xing S, Shen S, Xu B, Li X, Huan T (2023). BUDDY: Molecular formula discovery via bottom-up MS/MS interrogation. Nat. Methods.

[44] Deng J (2014). P-Terphenyl O-β-glucuronides, DNA topoisomerase inhibitors from *Streptomyces* sp. LZ35ΔgdmAI. Bioorg. Med. Chem. Lett..

[45] Yamazoe A, Hayashi KI, Kuboki A, Ohira S, Nozaki H (2004). The isolation, structural determination, and total synthesis of terfestatin A, a novel auxin signaling inhibitor from *Streptomyces* sp.. Tetrahedron Lett..

[46] Álvarez-Álvarez R (2015). Molecular genetics of naringenin biosynthesis, a typical plant secondary metabolite produced by *Streptomyces clavuligerus*. Microb. Cell Fact..

[47] AbuSara NF (2019). Comparative genomics and metabolomics analyses of clavulanic acid-producing *Streptomyces* species provides insight into specialized metabolism. Front. Microbiol..

[48] Shaikh AA, Nothias LF, Srivastava SK, Dorrestein PC, Tahlan K (2021). Specialized metabolites from ribosome engineered strains of *Streptomyces clavuligerus*. Metabolites.

[49] Clinger JA (2021). Structure and function of a dual reductase-dehydratase enzyme system involved in p-terphenyl biosynthesis. ACS Chem. Biol..

[50] Gui M, Zhang M-X, Wen-hui W, Sun P (2019). Natural occurrence, bioactivity and biosynthesis of elaiophylin analogues. Molecules.

[51] Lee, S. Y. *et al.* Structure determination and biological activities of elaiophylin produced by *Streptomyces* sp. MCY-846. *J. Microbiol. Biotechnol.* **6**, 245–249. Preprint at (1996).

[52] Klassen JL, Lee SR, Poulsen M, Beemelmanns C, Kim KH (2019). Efomycins K and L from a termite-associated *Streptomyces* sp. M56 and their putative biosynthetic origin. Front. Microbiol..

[53] Han Y (2016). Halichoblelide D, a new elaiophylin derivative with potent cytotoxic activity from mangrove-derived *Streptomyces* sp. 219807. Molecules.

[54] Sheng Y (2015). Identification of Elaiophylin Skeletal variants from the Indonesian *Streptomyces* sp. ICBB 9297. J. Nat. Prod..

[55] Wu C (2013). Identification of Elaiophylin Derivatives from the Marine-Derived Actinomycete *Streptomyces* sp. 7-145 using PCR-based screening. J. Nat. Prod..

[56] Supong K (2016). Antimicrobial compounds from endophytic *Streptomyces* sp. BCC72023 isolated from rice (*Oryza sativa* L.). Res. Microbiol..

[57] Burkhardt K, Fiedler H-P, Grabley S, Thiericke R, Zeeck A (1996). New Cineromycins and Musacins obtained by metabolite pattern analysis of *Streptomyces griseoviridis* (FH-S 1832). I. Taxonomy, fermentation, isolation and biological activity. J. Antibiot..

[58] Schneider A (1996). New Cineromycins and Musacins obtained by metabolite pattern analysis of *Streptomyces griseoviridis* FH-S 1832). II. Structure Elucidation. J. Antibiot..

[59] Fukushima T, Tanaka M, Gohbara M, Fujimori T (1998). Phytotoxicity of three lactones from *Nigrospora sacchari*. Phytochemistry.

[60] Ivanova V, Schlegel R, Dornberger K (1998). N′-methylniphimycin, a novel minor congener of niphimycin from *Streptomyces* spec. 57-13. J. Basic Microbiol..

[61] Chen Y (2022). Discovery of Niphimycin C from *Streptomyces yongxingensis* sp. nov. as a promising agrochemical fungicide for controlling banana fusarium wilt by destroying the mitochondrial structure and function. J. Agric. Food Chem..

[62] Hu Y (2018). Identification and proposed relative and absolute configurations of Niphimycins C-E from the marine-derived *Streptomyces* sp. IMB7-145 by genomic analysis. J. Nat. Prod..

[63] Usuki Y (2006). Structure-activity relationship studies on niphimycin, a guanidylpolyol macrolide antibiotic. Part 1: The role of the N-methyl-N″-alkylguanidinium moiety. Bioorg. Med. Chem. Lett..

[64] Takesako K, Beppu T (1984). Studies on new antifungal antibiotics, guanidylfungins A and B. I. Taxonomy, fermentation, isolation and characterization. J. Antibiot..

[65] Karki S (2010). The methoxymalonyl-acyl carrier protein biosynthesis locus and the nearby gene with the β-ketoacyl synthase domain are involved in the biosynthesis of galbonolides in *Streptomyces galbus*, but these loci are separate from the modular polyketide synt. FEMS Microbiol. Lett..

[66] Liu C, Zhang J, Lu C, Shen Y (2015). Heterologous expression of galbonolide biosynthetic genes in *Streptomyces coelicolor*. Antonie van Leeuwenhoek, Int. J. General Mol. Microbiol..

[67] Zhang J, Chang X, Li Y, Lu C (2016). Galbonolides from *Streptomyces* sp. SR107. Nat. Prod. Commun..

[68] Salituro GM (1995). Meridamycin: A novel nonimmunosuppressive FKBP12 ligand from *Streptomyces hygroscopicus*. Tetrahedron Lett..

[69] Gollan PJ, Bhave M, Aro EM (2012). The FKBP families of higher plants: Exploring the structures and functions of protein interaction specialists. FEBS Lett..

[70] Xiong Y, Sheen J (2014). The role of target of rapamycin signaling networks in plant growth and metabolism. Plant Physiol..

[71] Xiong F (2016). Tomato FK506 binding protein 12KD (FKBP12) mediates the interaction between rapamycin and target of rapamycin (TOR). Front. Plant. Sci..

[72] Montané MH, Menand B (2013). ATP-competitive mTOR kinase inhibitors delay plant growth by triggering early differentiation of meristematic cells but no developmental patterning change. J. Exp. Bot..

[73] Liu M, Lu C, Shen Y (2016). Four new meridamycin congeners from: *Streptomyces* sp. SR107. RSC Adv..

[74] He M, Haltli B, Summers M, Feng X, Hucul J (2006). Isolation and characterization of meridamycin biosynthetic gene cluster from *Streptomyces* sp. NRRL 30748. Gene.

[75] Sun Y (2006). Organization of the biosynthetic gene cluster in *Streptomyces* sp. DSM 4137 for the novel neuroprotectant polyketide meridamycin. Microbiology.

[76] Natsume M, Tashiro N, Doi A, Nishi Y, Kawaide H (2017). Effects of concanamycins produced by *Streptomyces scabies* on lesion type of common scab of potato. J. General Plant Pathol..

[77] Igarashi Y, Iida T, Yoshida R, Furumai T (2002). Pteridic acids A and B, novel plant growth promoters with auxin-like activity from *Streptomyces hygroscopicus* TP-A0451. J. Antibiot..

[78] Yang, Z. *et al. Streptomyces* alleviate abiotic stress in plant by producing pteridic acids. *bioRxiv* 2022.11.18.517137 (2022). 10.1038/s41467-023-43177-3 PMC10652019 37968347

[79] Buell CR (2003). The complete genome sequence of the *Arabidopsis* and tomato pathogen *Pseudomonas syringae* pv. tomato DC3000. Proc. Natl. Acad. Sci..

[80] Russell DW, Sambrook J (2001). Molecular Cloning: A Laboratory Manual.

[81] Chambers MC (2012). A cross-platform toolkit for mass spectrometry and proteomics. Nat. Biotechnol..

[82] Pluskal T, Castillo S, Villar-Briones A, Orešič M (2010). MZmine 2: Modular framework for processing, visualizing, and analyzing mass spectrometry-based molecular profile data. BMC Bioinf..

[83] Shannon P (2003). Cytoscape: A software environment for integrated models of biomolecular interaction networks. Genome Res..

[84] Ruttkies C, Schymanski EL, Wolf S, Hollender J, Neumann S (2016). MetFrag relaunched: Incorporating strategies beyond in silico fragmentation. J. Cheminform..

[85] Dührkop K, Shen H, Meusel M, Rousu J, Böcker S (2015). Searching molecular structure databases with tandem mass spectra using CSI:FingerID. Proc. Natl. Acad. Sci. USA.

[86] Loria R (1995). Differential production of thaxtomins by pathogenic *Streptomyces* species in vitro. Phytopathology.

[87] Blin K (2023). antiSMASH 7.0: New and improved predictions for detection, regulation, chemical structures and visualisation. Nucleic Acids Res..

[88] Navarro-Muñoz JC (2020). A computational framework to explore large-scale biosynthetic diversity. Nat Chem Biol.

[89] Gilchrist CLM, Chooi YH (2021). Clinker & clustermap.js: Automatic generation of gene cluster comparison figures. Bioinformatics.

[90] van den Belt M (2023). CAGECAT: The CompArative GEne cluster analysis toolbox for rapid search and visualisation of homologous gene clusters. BMC Bioinform..

[91] Harrison KJ, Crécy-Lagard VD, Zallot R (2018). Gene graphics: A genomic neighborhood data visualization web application. Bioinformatics.

[92] de Mendiburu, F. Agricolae: Statistical procedures for agricultural research. Preprint at (2020).

[93] Wickham H (2016). Ggplot2: Elegant Graphics for Data Analysis.

[94] Ahlmann-Eltze, C. ggsignif: Significance Brackets for ‘ggplot2’. Preprint at (2019).

[95] Pedersen, T. L. Patchwork: The composer of plots. Preprint at (2020).

