# Supplementary material for: Exploring the specialized metabolome of the plant pathogen Streptomyces sp. 11-1-2
Source: Sci Rep. 2024 May 6;14:10414. doi: 10.1038/s41598-024-60630-5 (PMC11074319; doi:10.1038/s41598-024-60630-5)
Supplement: Supplementary file 1 — Supplementary Information. [file 41598_2024_60630_MOESM1_ESM.docx]

Supporting information for

# EXPLORING THE SPECIALIZED METABOLOME OF THE PLANT PATHOGEN *STREPTOMYCES* SP. 11-1-2

Gustavo A. Díaz-Cruz† and Dawn R.D. Bignell*

Department of Biology, Memorial University of Newfoundland, St. John’s, NL, Canada

* Address correspondence to: dbignell@mun.ca; Tel. +01-709-864-4573

† Current address: Plant Protection Research Center (CIPROC), Phytopathology Department, Agronomy School, Universidad de Costa Rica, San Jose, Costa Rica


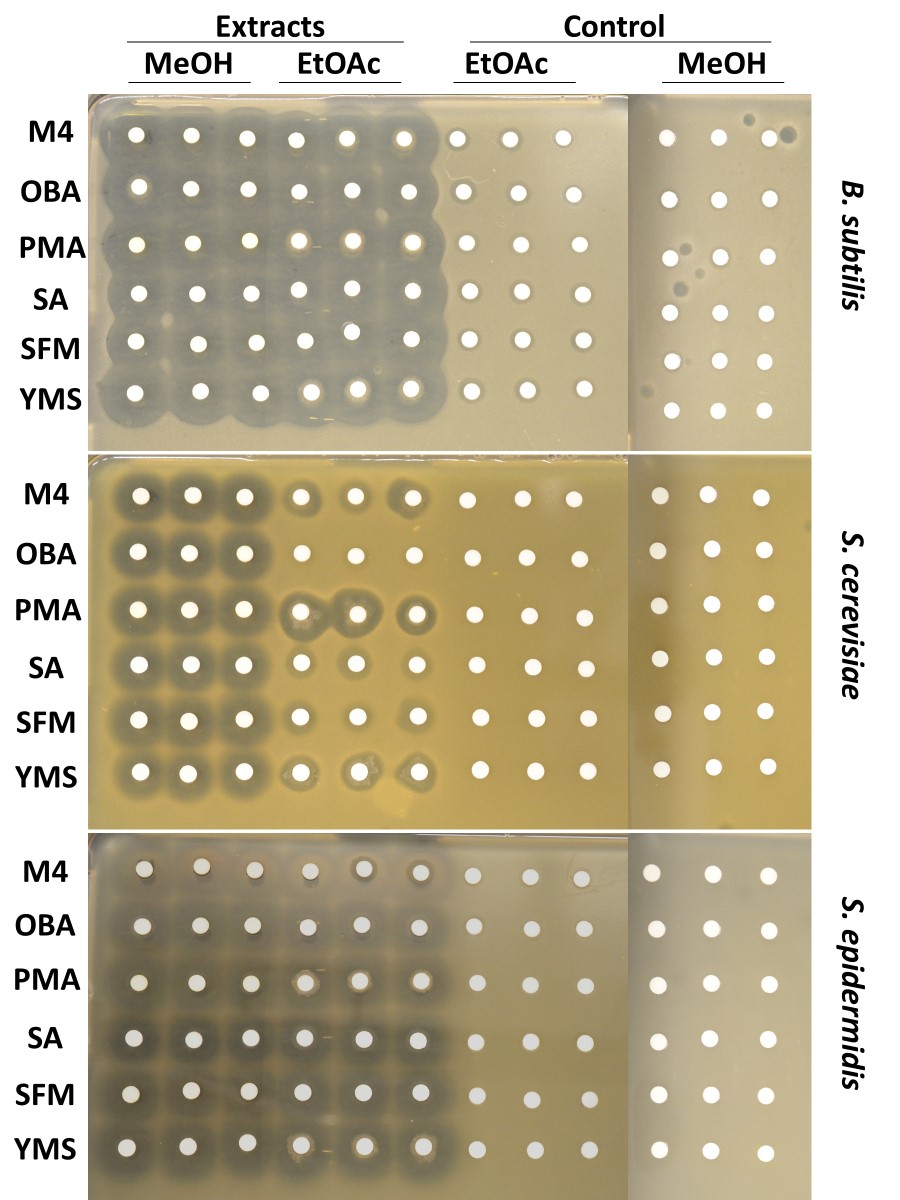


**Figure S1**. Antimicrobial bioassay using organic culture extracts against three different indicator organisms. The assay was also performed using the indicator organisms *E. coli* and *P. syringae* pv.*tomato* DC3000, but no inhibition was detected.

**
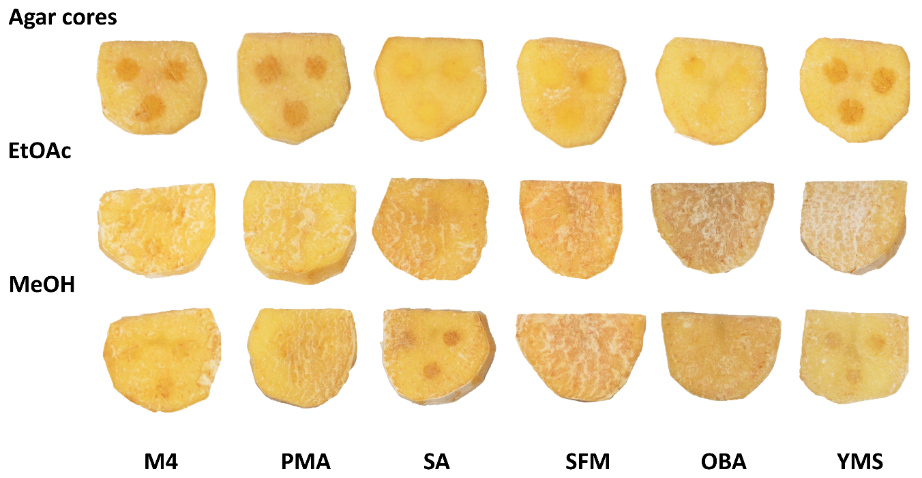
**

**Figure S2**. Control slices for the potato tuber slice assays using agar cores and organic culture extracts.


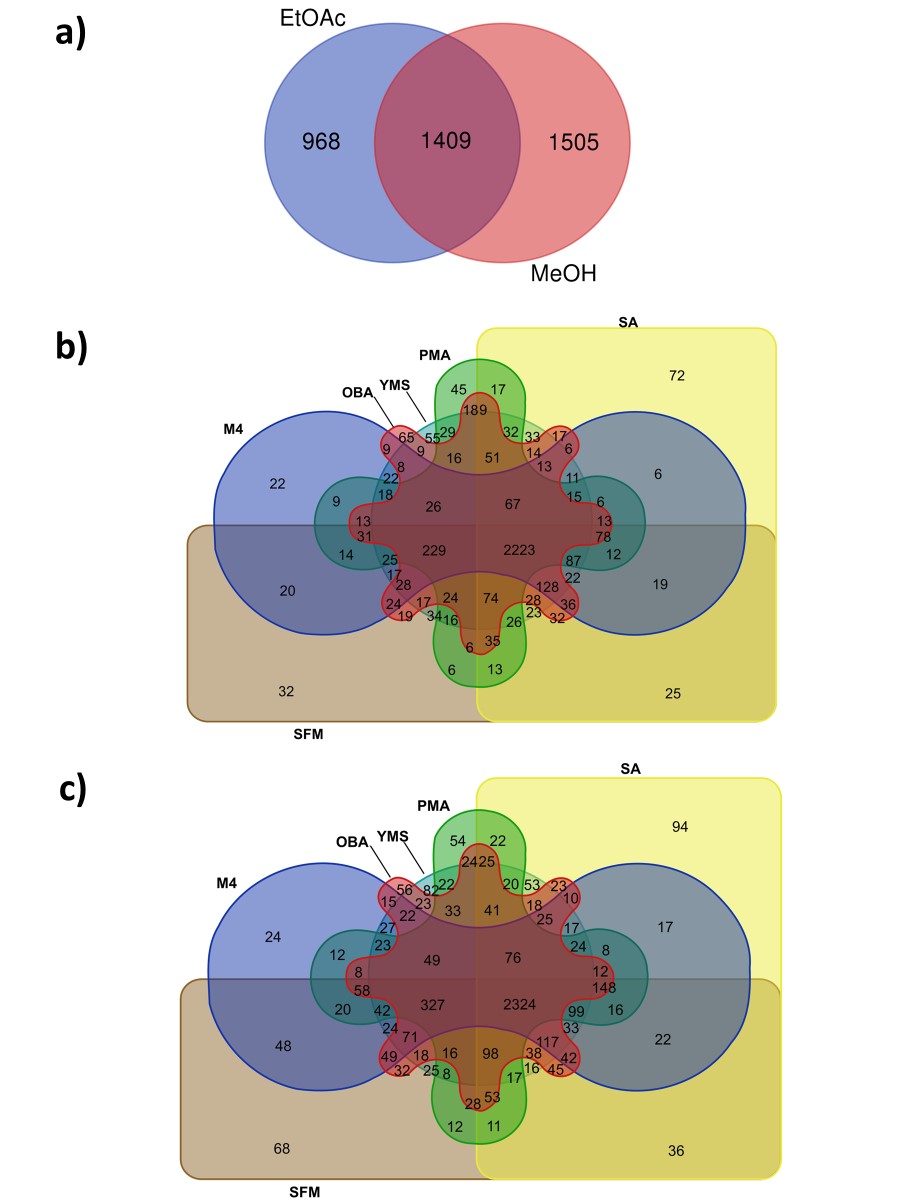


**Figure S3**. Venn diagrams showing the number of shared features detected by the IIMN analysis. The comparison was made by a) organic solvent and by media for b) EtOAc and c) MeOH extracts. The diagram was prepared using the precursor mass obtained from the quantification table after processing in the GNPS website.


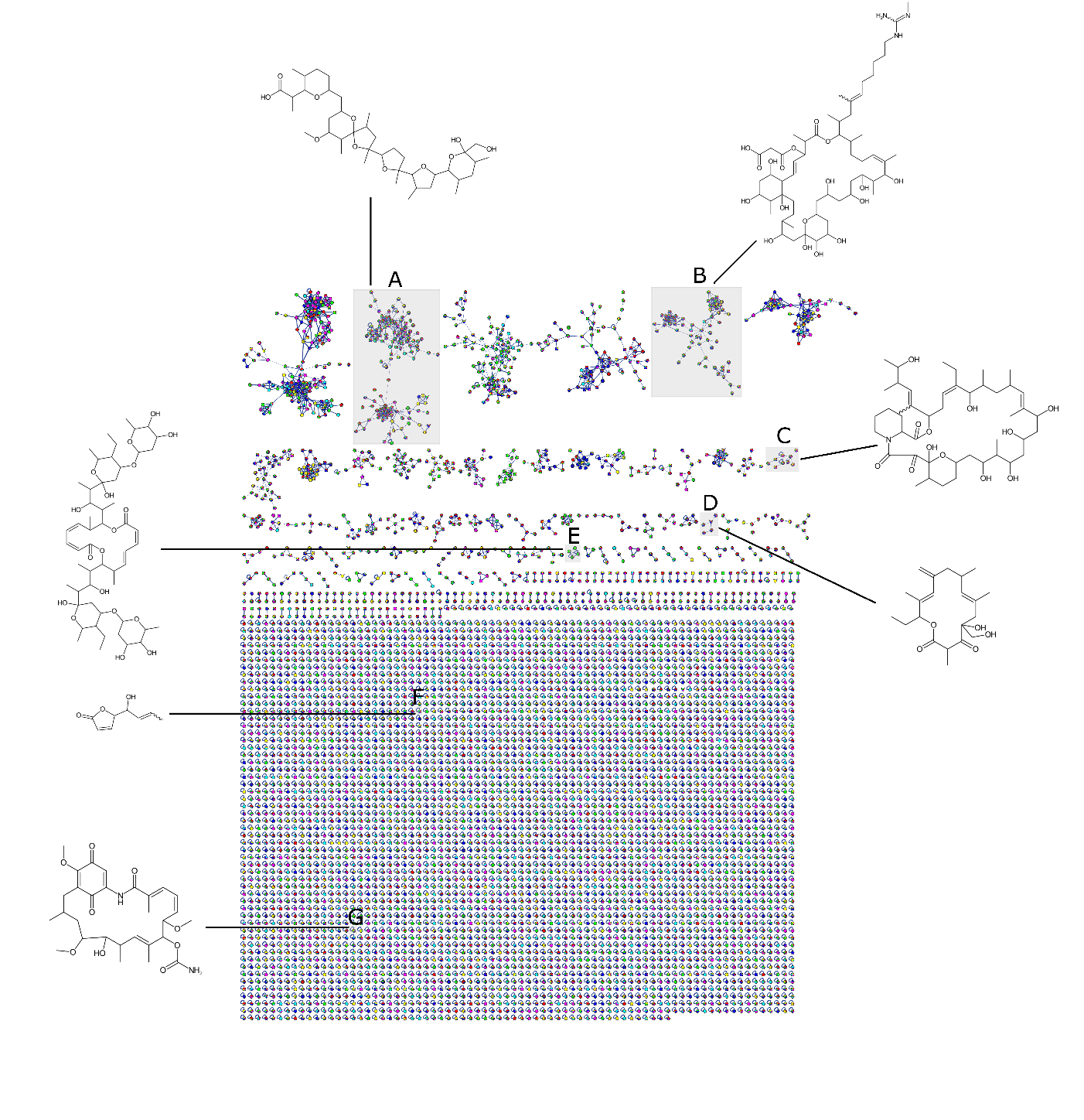


**Figure S4**. Ion identity molecular networks of *Streptomyces* sp. 11-1-2 metabolites. The metabolites were extracted from M4, OBA, PMA, SA, SFM and YMS plate cultures using MeOH and were analyzed using untargeted LC-MS/MS in both positive and negative ionization mode. Each node in the networks represents one fragmentation spectrum, and nodes are linked if the cosine score is >0.7 and there are at least 6 matched fragment ions. Networks containing selected specialized metabolites are highlighted and numbered based on the main metabolite: A, nigericin; B, guanidylfungin A; C, meridamycins; D, galbonolide B; E, elaiophylin; F, musacin D; G, geldanamycin.


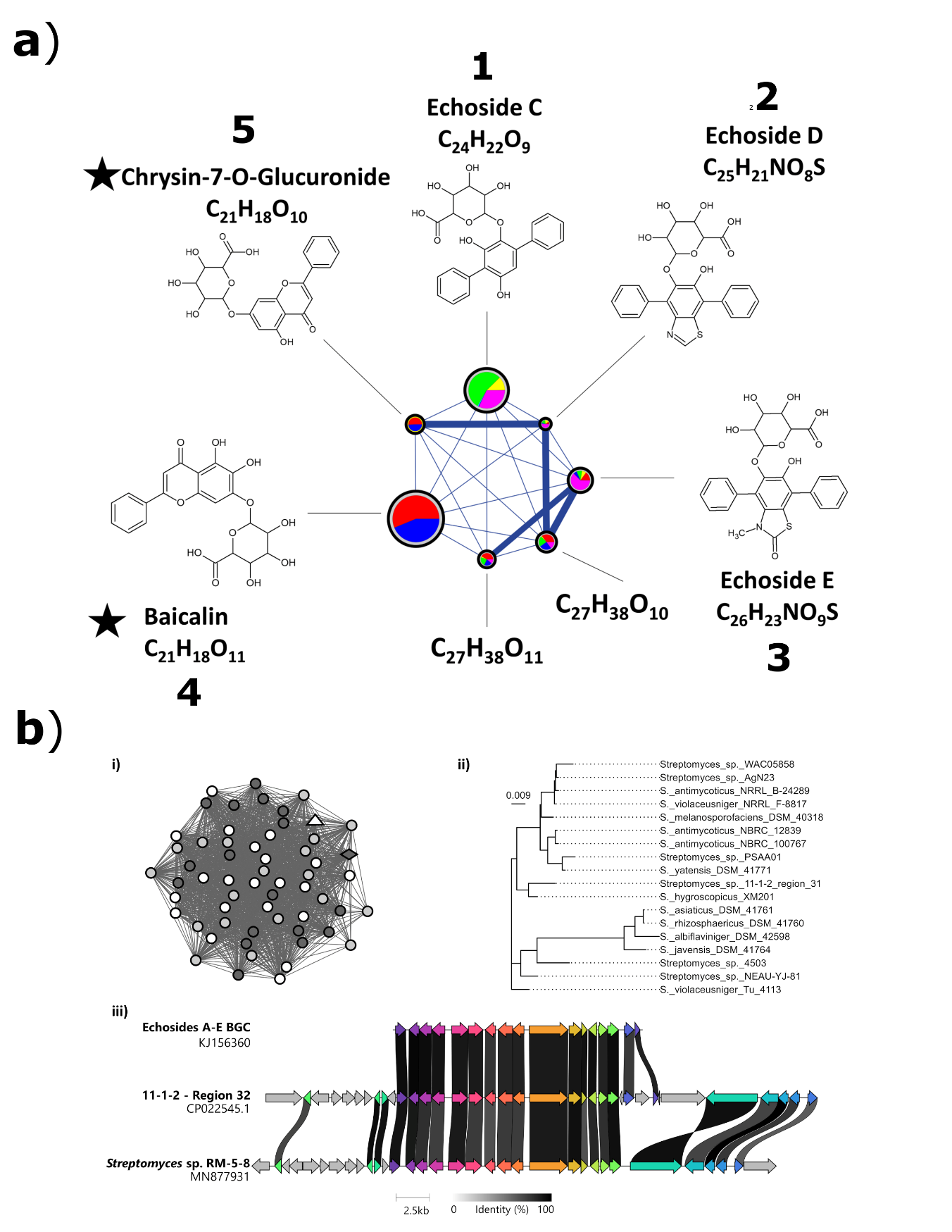


**Figure S5**. a) Ion identity molecular network (negative ion mode) for echoside metabolites in the *Streptomyces* sp. 11-1-2 EtOAc culture extracts. Each node in the network represents a one fragmentation spectrum, and the predicted molecular formula and structure of each is shown. ⋆ indicates metabolites with a match to the GNPS spectral library. Nodes are linked if the cosine score is >0.7 and there are at least 6 matched fragment ions, and the thickness of the line increases at higher cosine values. The size of the node is relative to the total abundance of each compound in the analyzed culture extracts. The pie charts represent the relative abundance of each compound in the six different culture extracts: Red = M4, Yellow = OBA, Light green = PMA, Teal = SA, Blue = SFM, Violet = YMS. b) Genomic analysis of region 32 in the 11-1-2 genome. i) BiG-SCAPE network. The diamond represents the 11-1-2 region 32, the triangle represents the MIBiG BGC, and the colour of the nodes represents different gene cluster families (GCFs). ii) Phylogenetic tree showing the GCF containing region 32 and closely related BGCs in the custom *Streptomyces* BGC database. iii) Alignment of the echoside A-E BGC available in the MIBIG database with a similar BGC from *Streptomyces* sp. RM-5-8 and the predicted echoside BGC at region 32 in *Streptomyces* sp. 11-1-2. Genes coloured the same belong to the same functional group, and homologues are linked by shaded areas that indicate the % amino acid identity of the corresponding protein products.


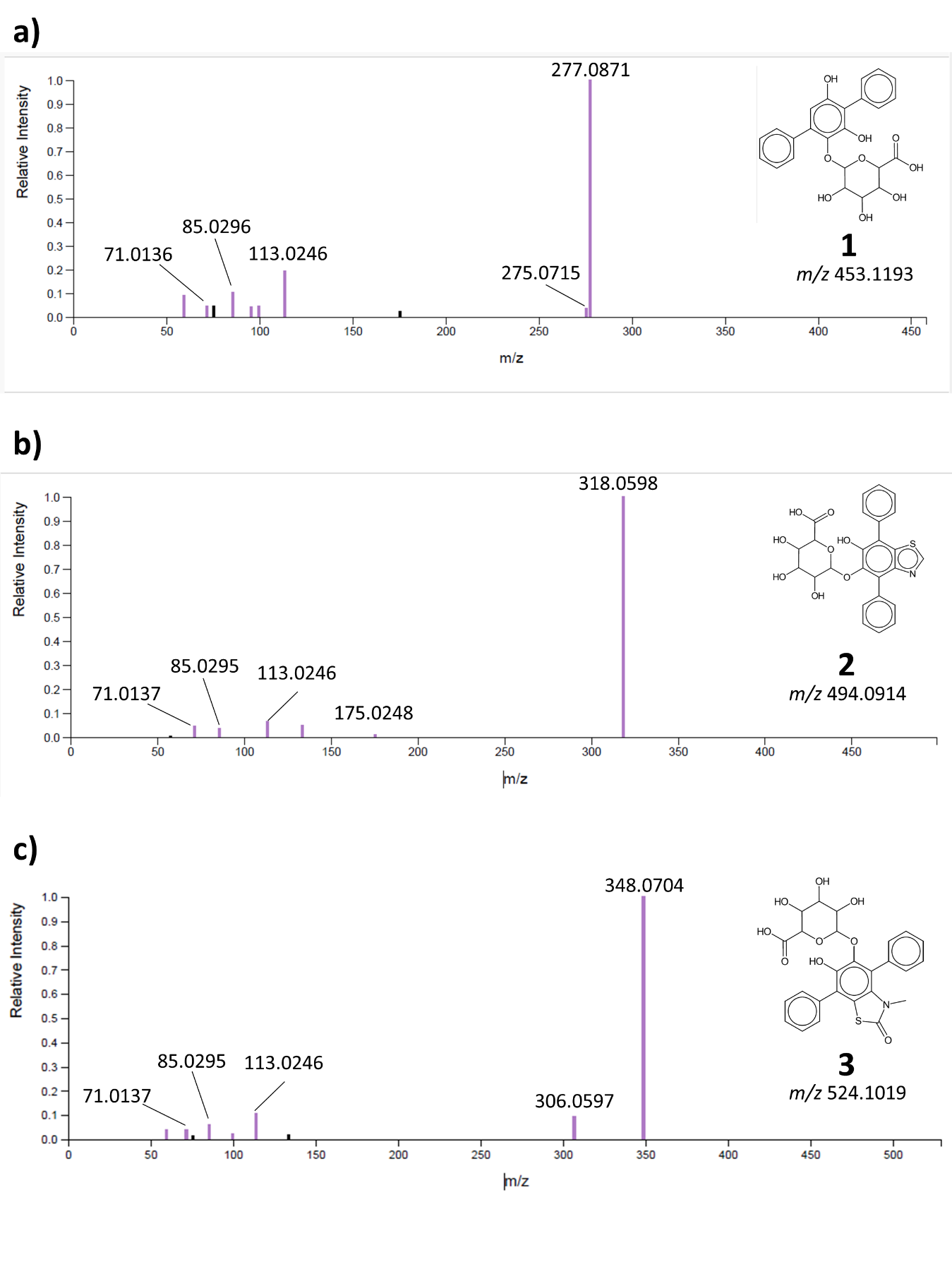


**Figure S6.** SIRIUS metabolite prediction for echosides. The LC-MS/MS fragmentation pattern for a) echoside C (**1**), b) echoside D (**2**) and c) echoside E (**3**) are shown. Each molecular structure is shown in the respective pattern as an inset. Peaks in purple represent peaks used for molecular formula and substructure annotation; peaks in green were used only for molecular formula prediction; peaks in black are noise or cannot be explained.


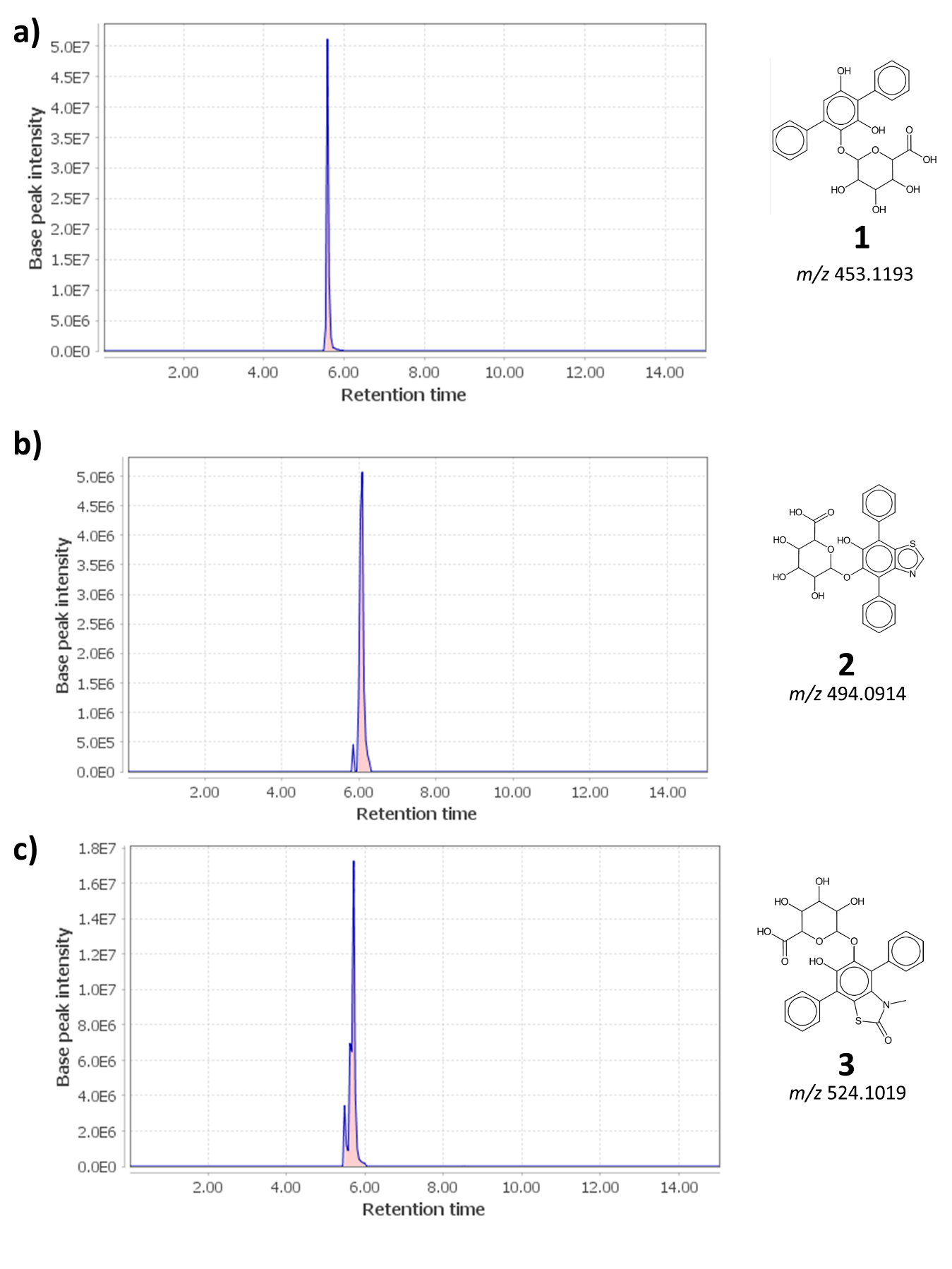


**Figure S7**. Representative extracted ion chromatogram for a) echoside C (**1**), b) echoside D (**2**) and c) echoside E (**3**) are shown. The peaks correspond to the *m/z* value calculated for each compound.


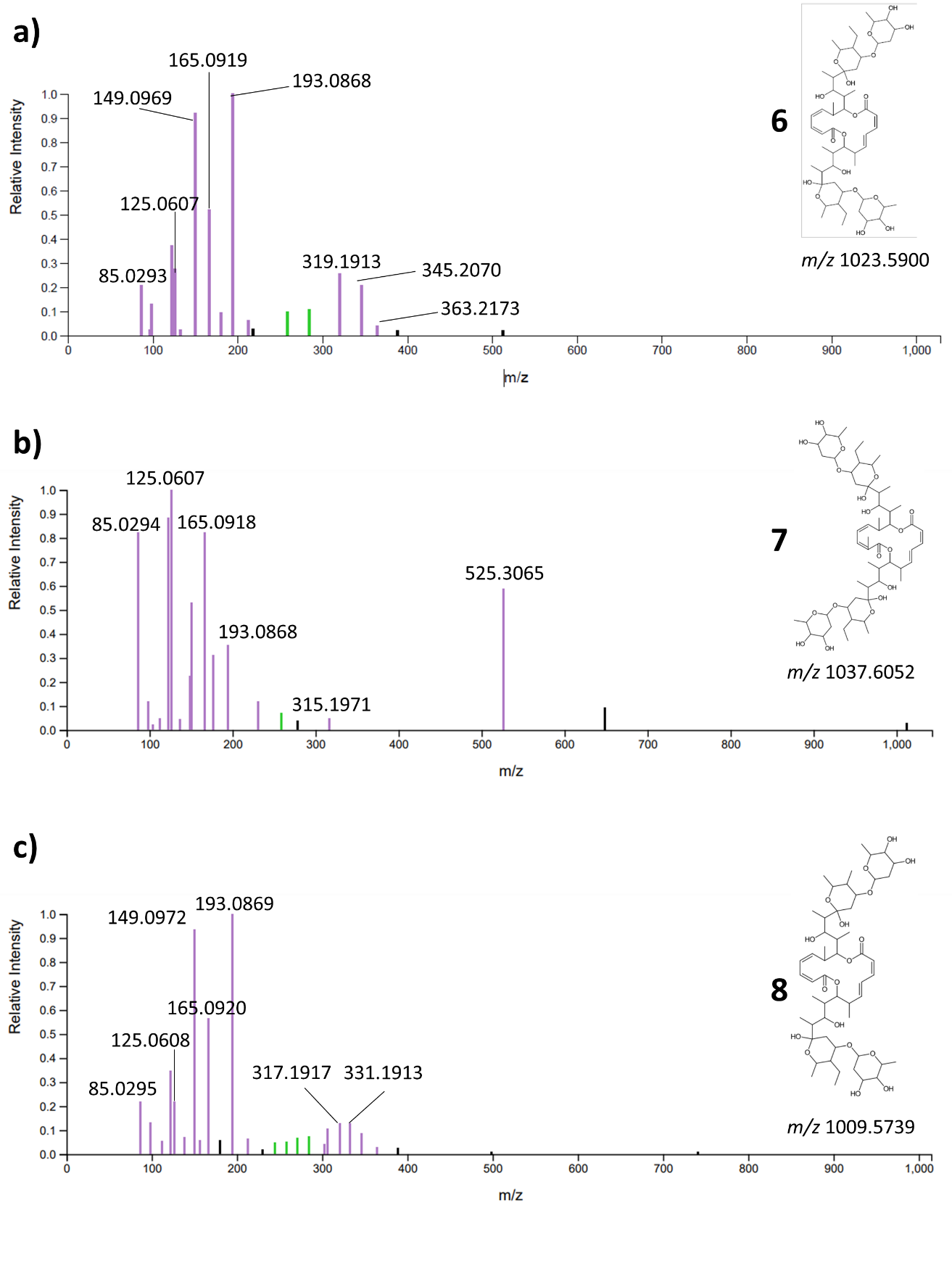


**Figure S8**. SIRIUS metabolite prediction for elaiophylins. The LC-MS/MS fragmentation pattern for a) elaiophylin (**6**), b) 2-methylelaiophylin (**7**) and c) efomycin G (**8**) are shown. Each molecular structure is shown in the respective pattern as an inset. Peaks in purple represent peaks used for molecular formula and substructure annotation; peaks in green were used only for molecular formula prediction; peaks in black are noise or cannot be explained.


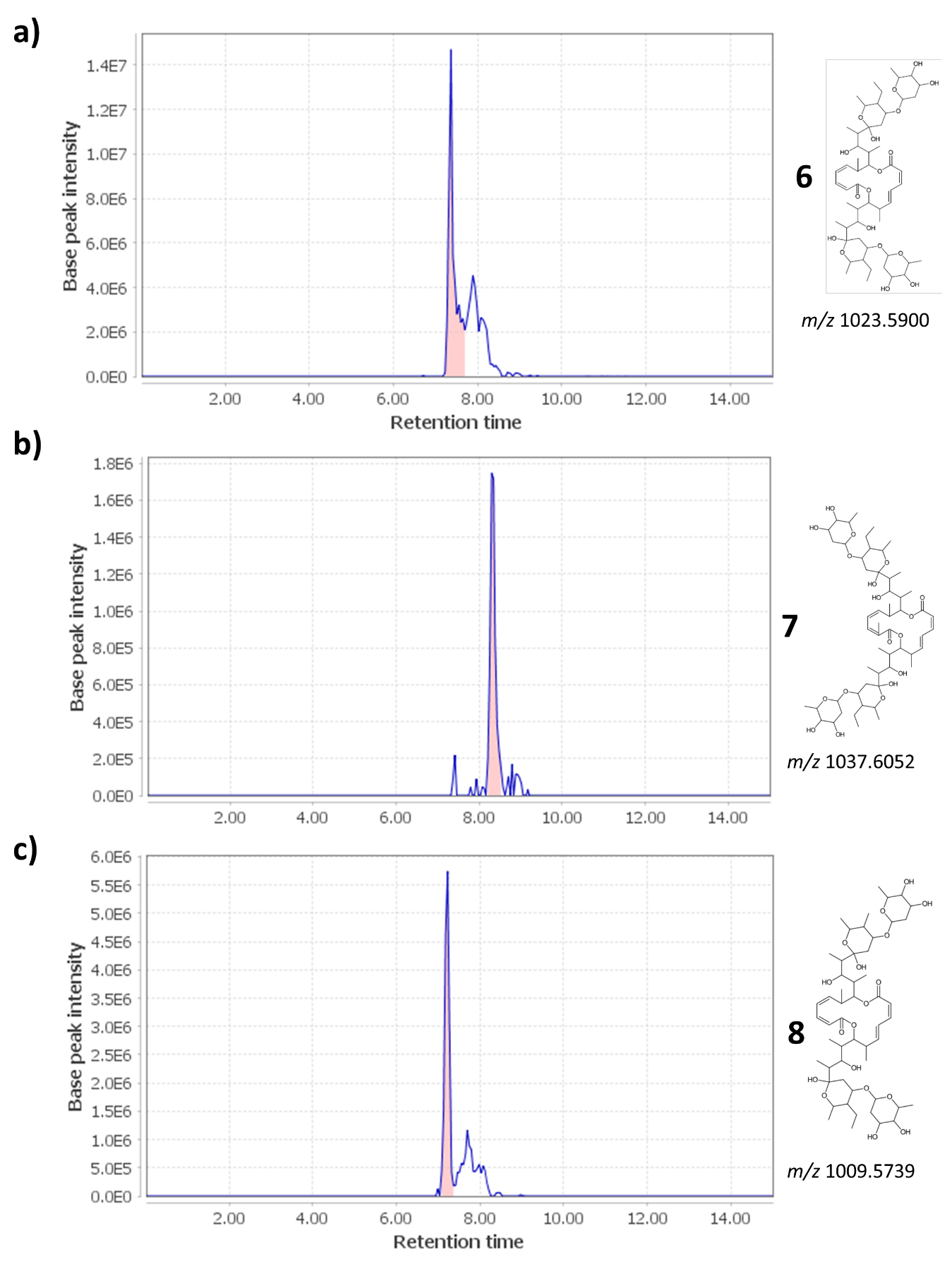


**Figure S9**. Representative extracted ion chromatogram for a) elaiophylin (**6**), b) 2-methylelaiophylin (**7**) and c) efomycin G (**8**) are shown. The peaks correspond to the *m/z* value calculated for each compound.


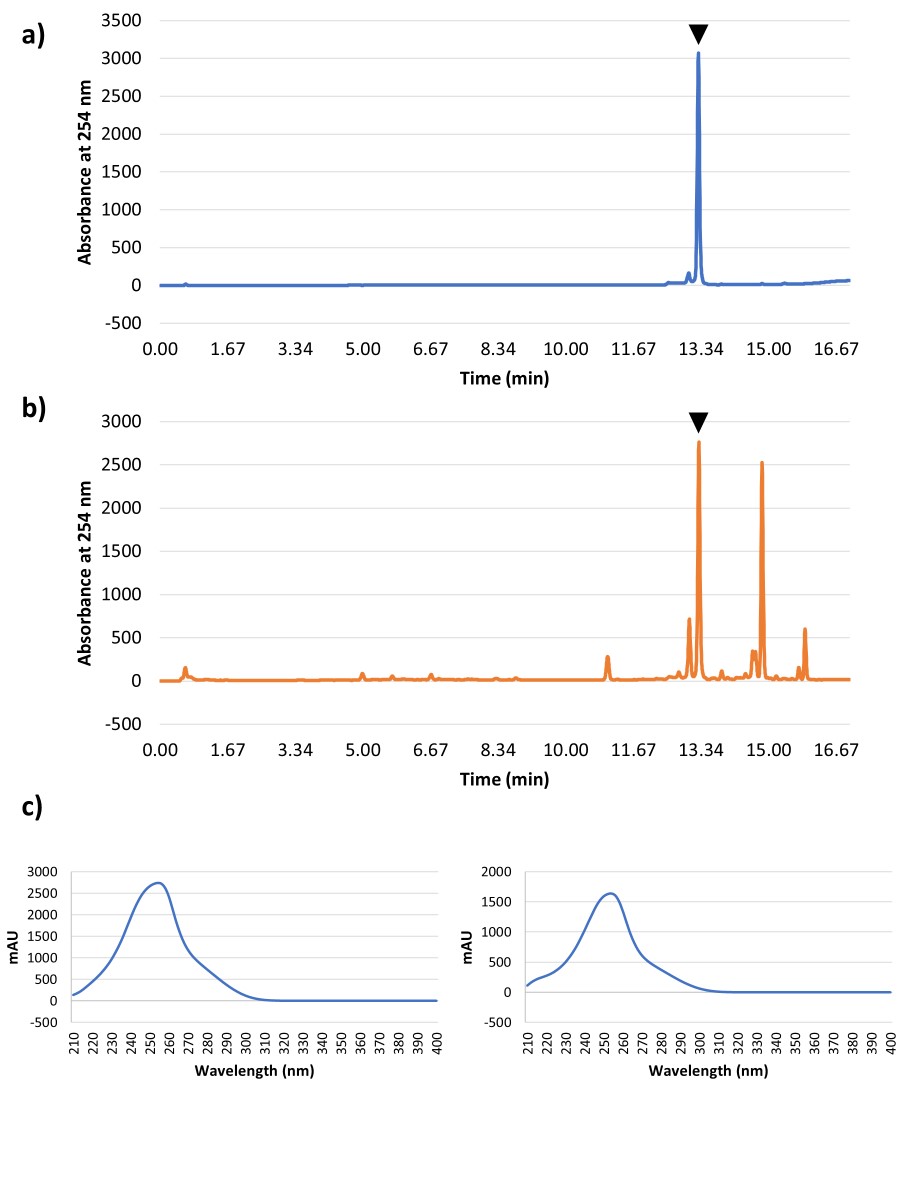


**Figure S10**. Elaiophylin detection using RP-HPLC. Shown are the chromatograms of a) the elaiophylin standard and b) a representative PMA organic extract. The inverted triangle indicates the peak corresponding to elaiophylin. c) Absorbance spectrum of the elaiophylin standard (left) and the elaiophylin peak from the PMA extract (right).


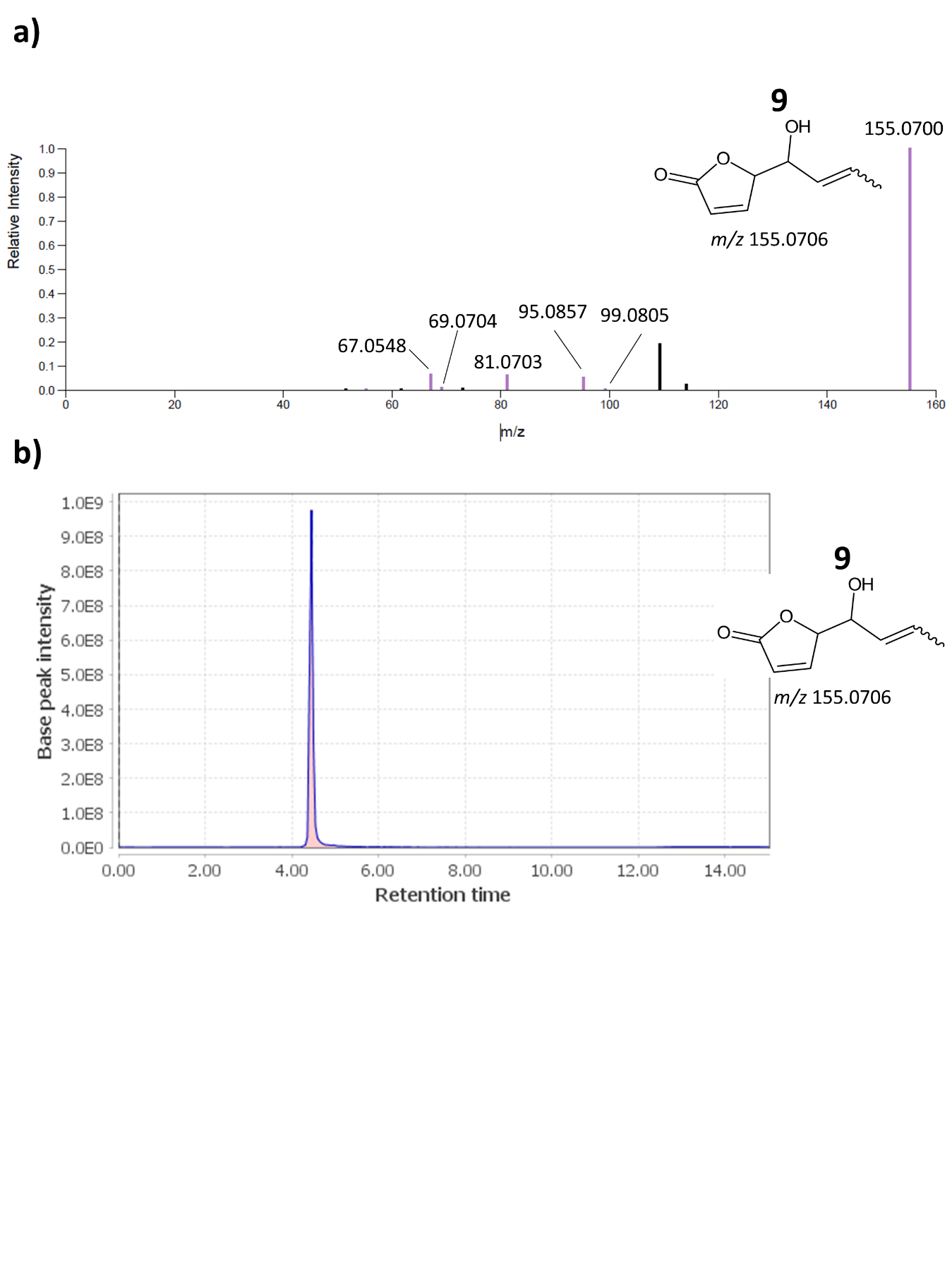


**Figure S11.** a) SIRIUS metabolite prediction for musacin D (**9**). The LC-MS/MS fragmentation pattern for musacin D is shown. The molecular structure is shown as an inset. Peaks in purple represent peaks used for molecular formula and substructure annotation; peaks in black are noise or cannot be explained. b) Representative extracted ion chromatogram for musacin D. The peak corresponds to the *m/z* value calculated for the compound.


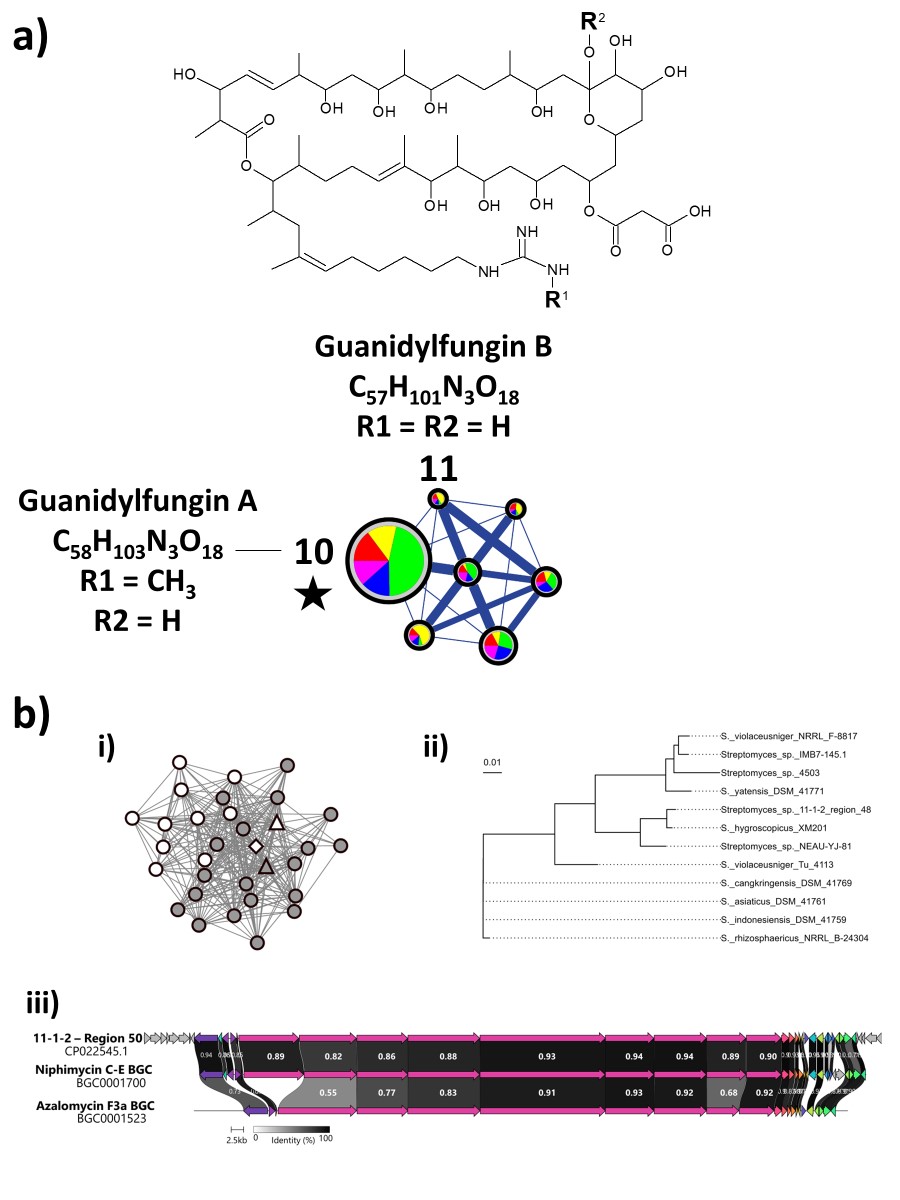


**Figure S12**. a) Ion identity molecular network (positive ion mode) for guanidylfungins in the *Streptomyces* sp. 11-1-2 EtOAc culture extracts. Each node in the network represents one fragmentation spectrum, and the predicted molecular formula and structure of each is shown. ⋆ indicates metabolites with a match to the GNPS spectral library. Nodes are linked if the cosine score is >0.7 and there are at least 6 matched fragment ions, and the thickness of the line increases at higher cosine values. The size of the node is relative to the total abundance of each compound in the analyzed culture extracts. The pie charts represent the relative abundance of each compound in the six different culture extracts: Red = M4, Yellow = OBA, Light green = PMA, Teal = SA, Blue = SFM, Violet = YMS. b) Genomic analysis of region 50 in the 11-1-2 genome. i) BiG-SCAPE network. The diamond represents the 11-1-2 region 50, the triangle represents the MIBiG BGC, and the colour of the nodes represents different gene cluster families (GCFs). ii) Phylogenetic tree showing the GCF containing region 50 and closely related BGCs in the custom *Streptomyces* BGC database. iii) Alignment of the niphimycin C-E and azalomycin F3a BGCs available in the MIBIG database with the predicted BGC at region 50 in *Streptomyces* sp. 11-1-2. Genes coloured the same belong to the same functional group, and homologues are linked by shaded areas that indicate the % amino acid identity of the corresponding protein products.


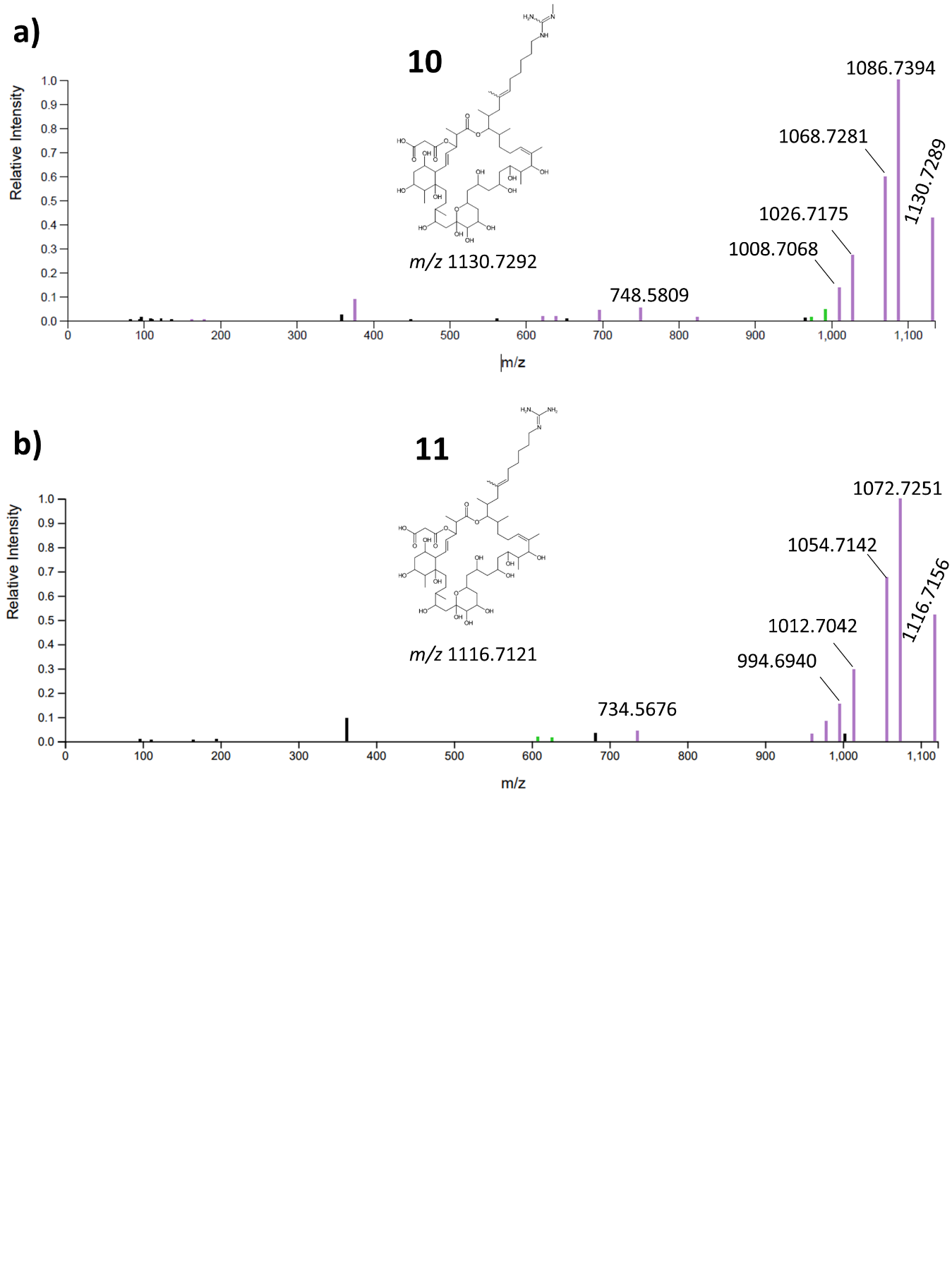


**Figure S13**. SIRIUS metabolite prediction for guanidylfungins. The LC-MS/MS fragmentation pattern for a) guanidylfungin A (**10**) and b) guanidylfungin B (**11**) are shown. Each molecular structure is shown in the respective pattern as an inset. Peaks in purple represent peaks used for molecular formula and substructure annotation; peaks in green were used only for molecular formula prediction; peaks in black are noise or cannot be explained.


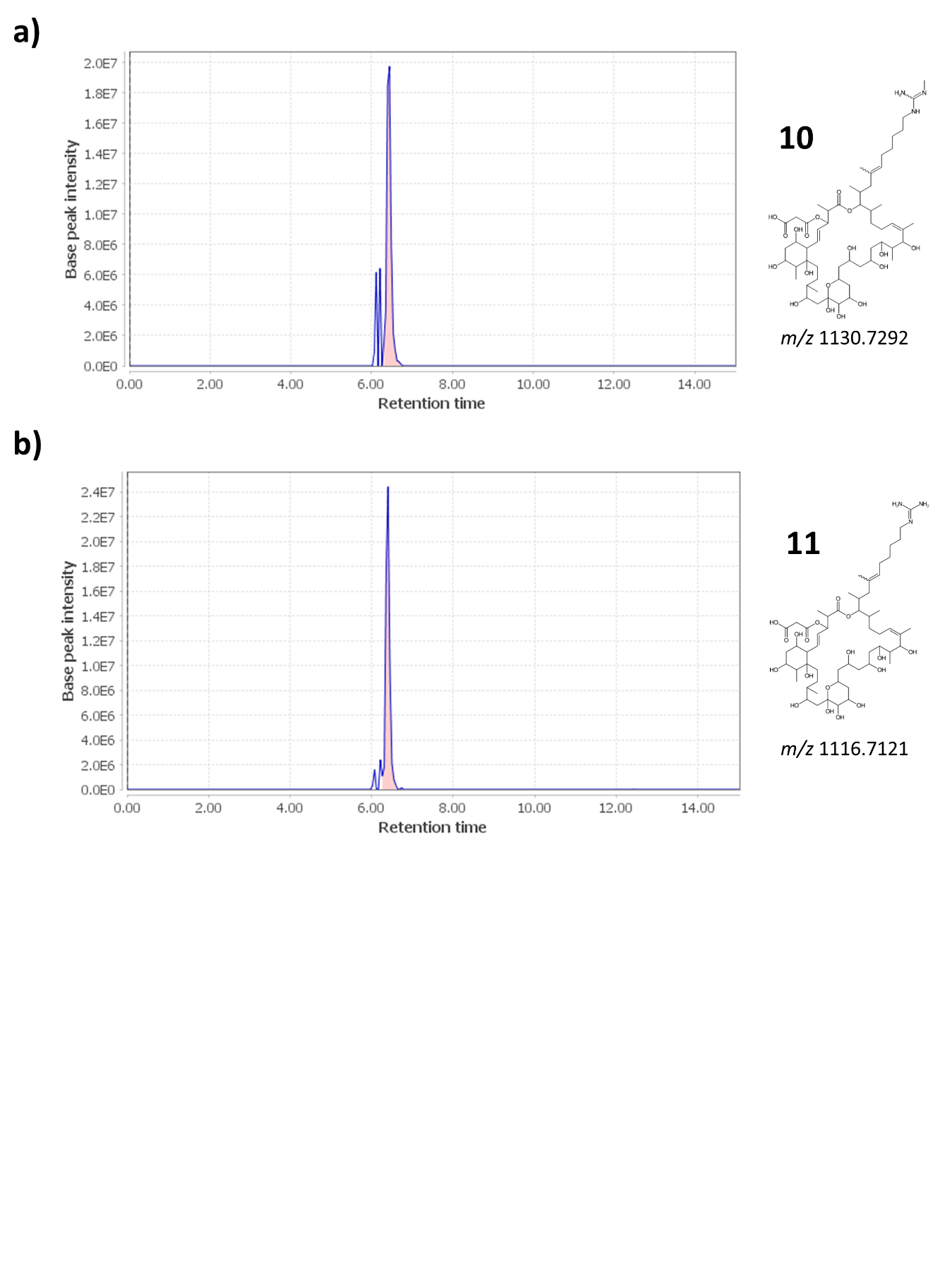


**Figure S14**. Representative extracted ion chromatogram for a) guanidylfungin A (**10**) and b) guanidylfungin B (**11**) are shown. The peaks correspond to the *m/z* value calculated for each compound.


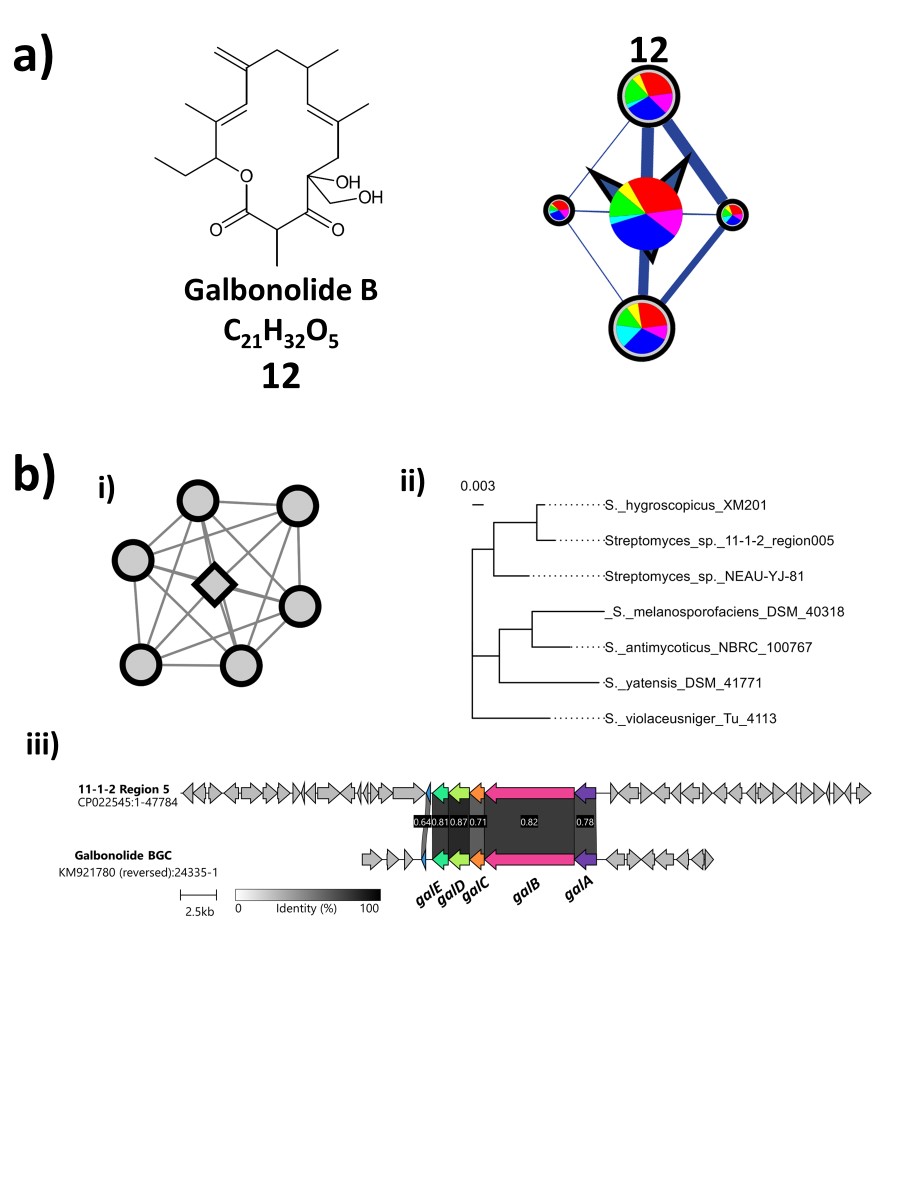


**Figure S15**. a) Ion identity molecular network (positive ion mode) for galbonolide B in the *Streptomyces* sp. 11-1-2 EtOAc culture extracts. Each node in the network represents one fragmentation spectrum, and the predicted molecular formula and structure of each is shown. A triangle behind a node represents a MS1 adduct. Nodes are linked if the cosine score is >0.7 and there are at least 6 matched fragment ions, and the thickness of the line increases at higher cosine values. The size of the node is relative to the total abundance of each compound in the analyzed culture extracts. The pie charts represent the relative abundance of each compound in the six different culture extracts: Red = M4, Yellow = OBA, Light green = PMA, Teal = SA, Blue = SFM, Violet = YMS. b) Genomic analysis of region 5 in the 11-1-2 genome. i) BiG-SCAPE network. The diamond represents the 11-1-2 region. ii) Phylogenetic tree showing closely related BGCs in the custom *Streptomyces* BGC database. iii) Alignment of the galbonolide BGC with the BGC predicted for the 11-1-2 region 5. Genes coloured the same belong to the same functional group, and homologues are linked by shaded areas that indicate the % amino acid identity of the corresponding protein products.


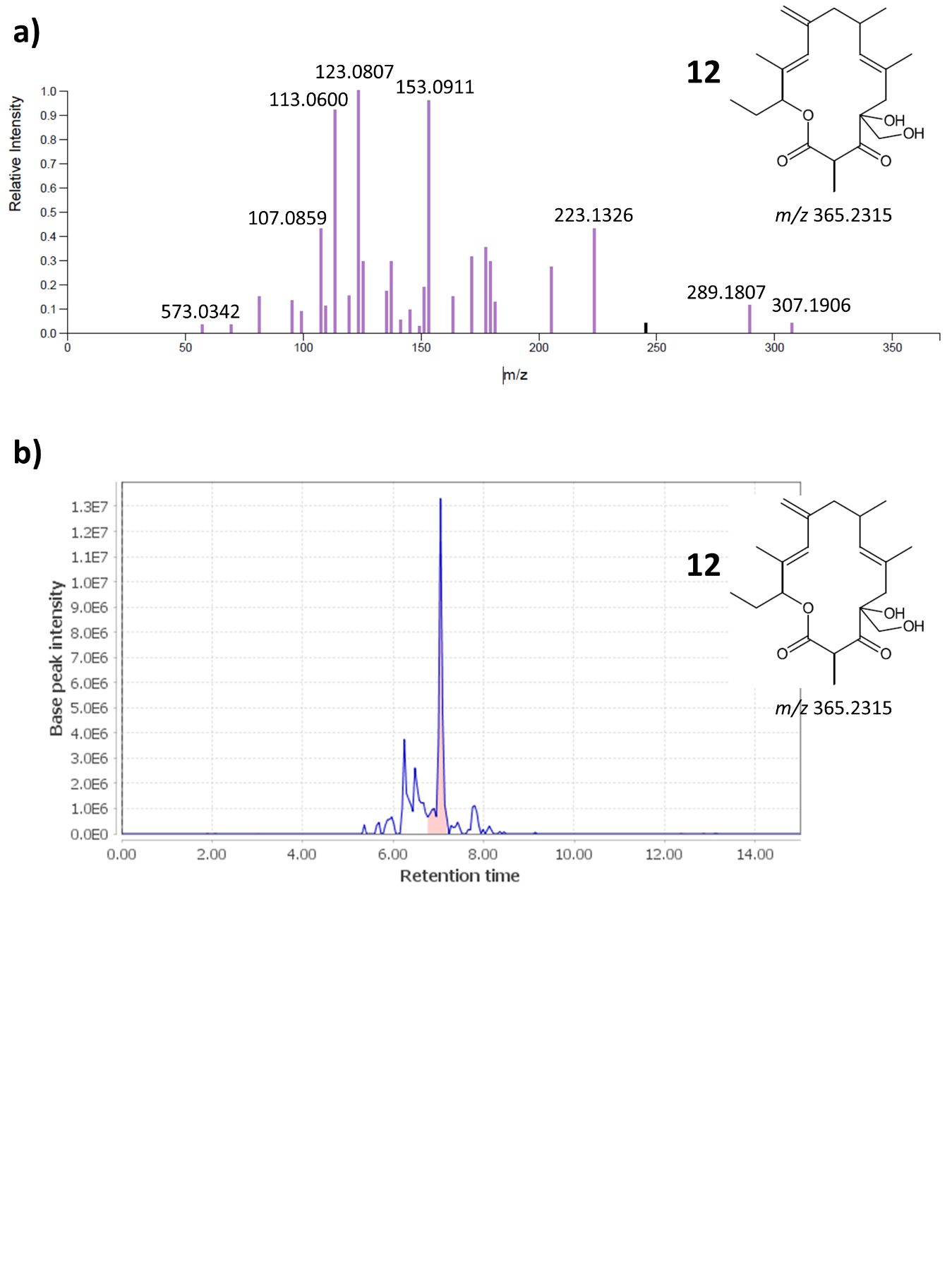


**Figure S16.** a) SIRIUS metabolite prediction for galbonolide B (**12**). The LC-MS/MS fragmentation pattern for galbonolide B is shown. The molecular structure is shown as an inset. Peaks in purple represent peaks used for molecular formula and substructure annotation; peaks in green were used only for molecular formula prediction; peaks in black are noise or cannot be explained. b) Representative extracted ion chromatogram galbonolide B. The peak corresponds to the *m/z* value calculated for the compound.


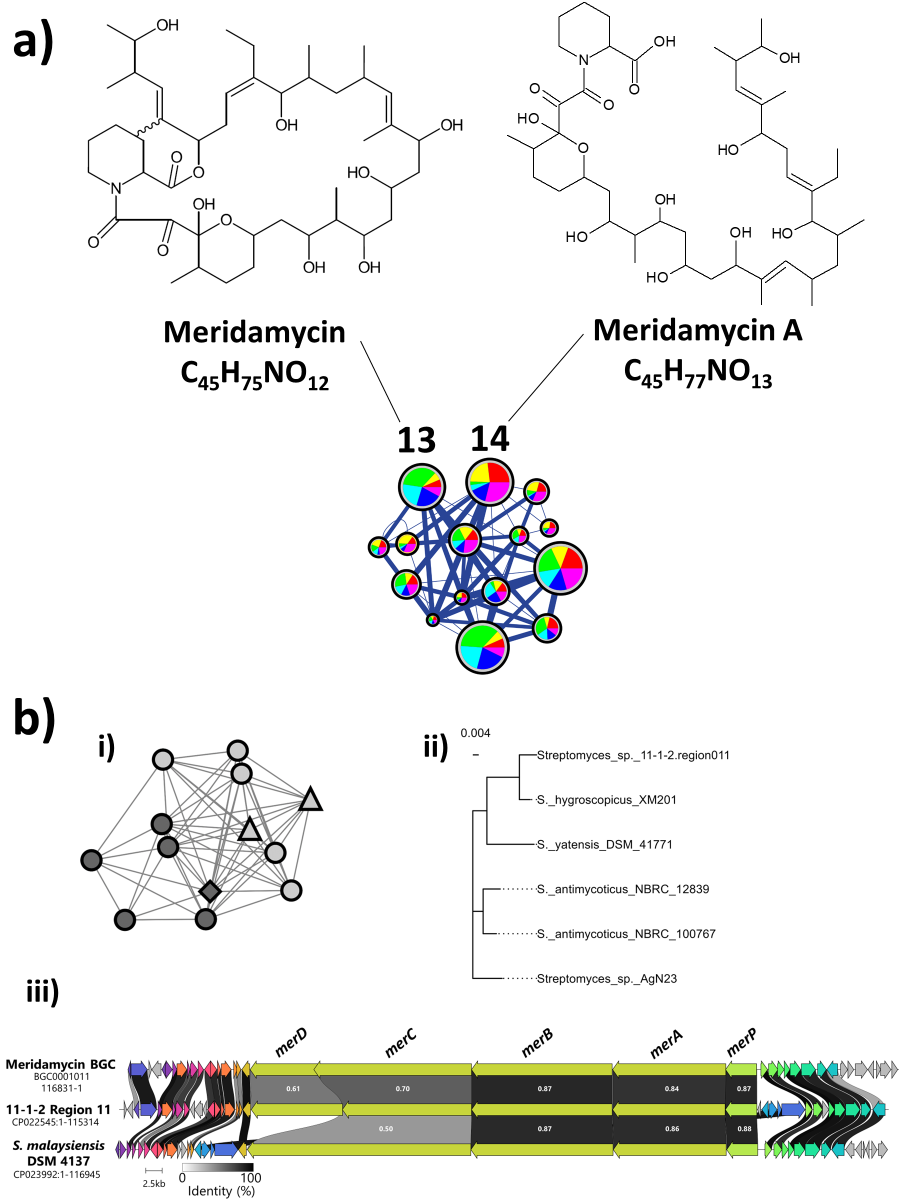


**Figure S17**. a) Ion identity molecular network (positive ion mode) for meridamycins in the *Streptomyces* sp. 11-1-2 EtOAc extracts. Each node in the network represents one fragmentation spectrum, and the predicted molecular formula and structure of each is shown. Nodes are linked if the cosine score is >0.7 and there are at least 6 matched fragment ions, and the thickness of the line increases at higher cosine values. The size of the node is relative to the total abundance of each compound in the analyzed culture extracts. The pie charts represent the relative abundance of each compound in the six different culture extracts: Red = M4, Yellow = OBA, Light green = PMA, Teal = SA, Blue = SFM, Violet = YMS. b) Genomic analysis of region 11 in the 11-1-2 genome. i) BiG-SCAPE network. The diamond represents the 11-1-2 region, the triangles represent the MIBiG BGCs, and the colour of the nodes represents the gene cluster family. ii) Phylogenetic tree showing the GCF containing region 11 and related BGCs in the custom *Streptomyces* BGC database. iii) Alignment of the meridamycin BGC, a similar cluster from *S. malaysiensis* DSM 4137 and the BGC predicted for the 11-1-2 region 11. Genes coloured the same belong to the same functional group, and homologues are linked by shaded areas that indicate the % amino acid identity of the corresponding protein products.


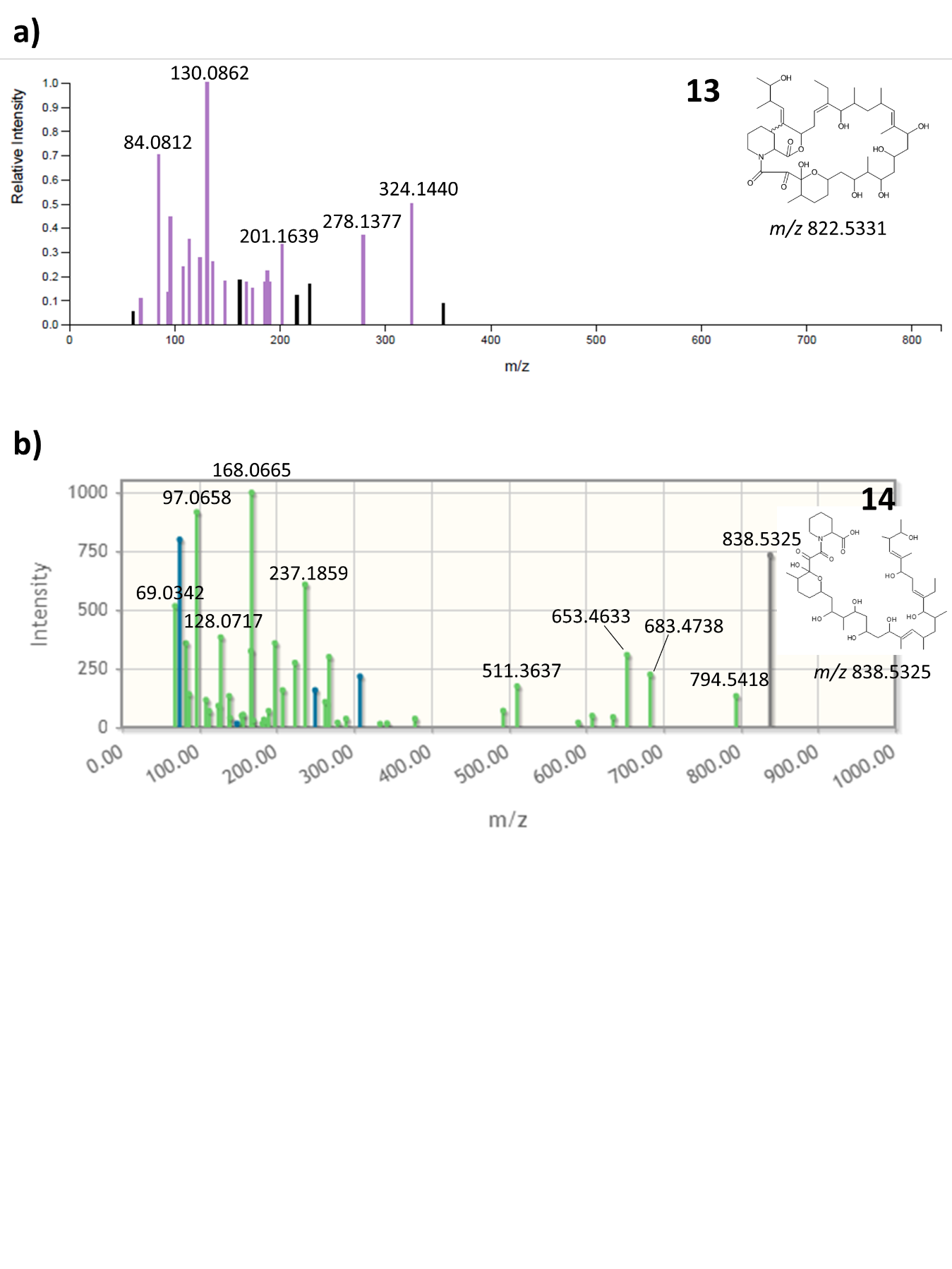


**Figure S18**. SIRIUS and MetFrag metabolite prediction for meridamycins. The LC-MS/MS fragmentation pattern for a) meridamycin (**13**) and b) meridamycin A (**14**) are shown. Each molecular structure is shown in the respective pattern as an inset. For meridamycin, the peaks in purple represent peaks used for molecular formula and substructure annotation; peaks in green were used only for molecular formula prediction; peaks in black are noise or cannot be explained. For meridamycin A, the MetFrag prediction shows green peaks as matches to the expected fragmentation pattern, while blue peaks did not match the expected pattern, and the gray peak is excluded from the analysis.


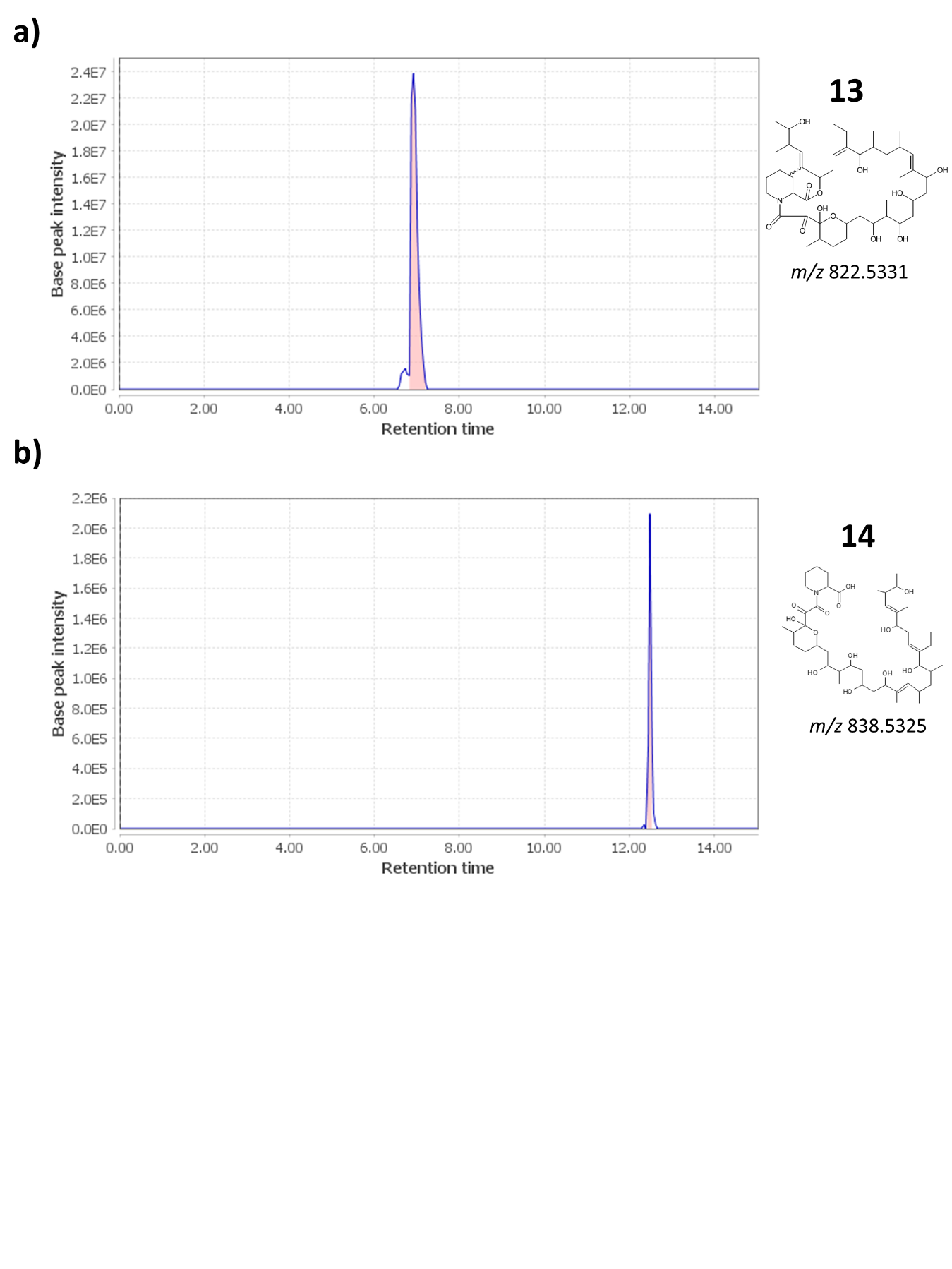


**Figure S19**. Representative extracted ion chromatogram for a) meridamycin (**13**) and b) meridamycin A (**14**) are shown. The peaks correspond to the *m/z* value calculated for each compound.


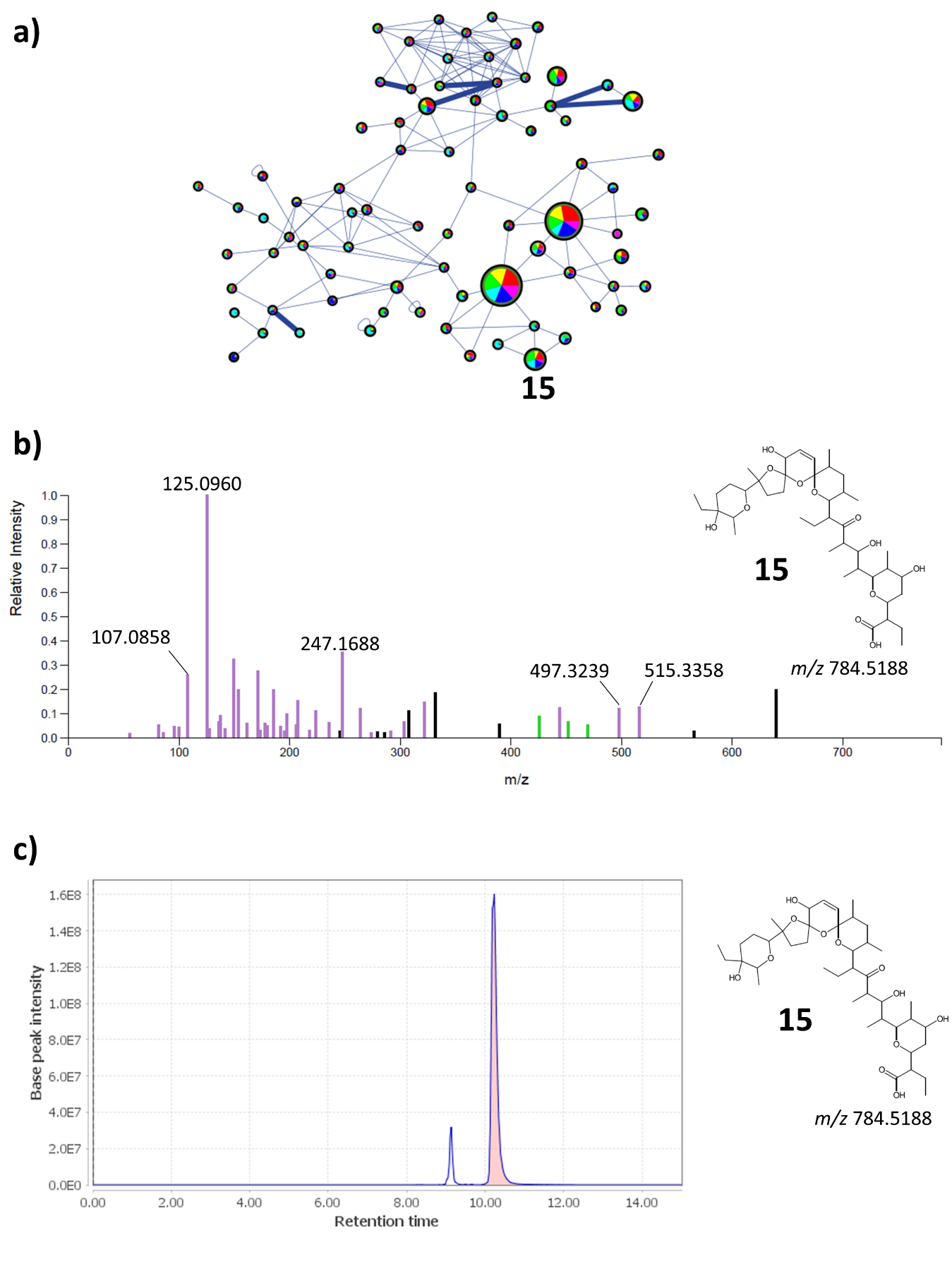


**Figure S20**. a) Nigericin network showing the nigericin-related compound **15**. b) SIRIUS metabolite prediction for **15**. The LC-MS/MS fragmentation pattern for the feature is shown. The molecular structure is shown as an inset. Peaks in purple represent peaks used for molecular formula and substructure annotation; peaks in green were used only for molecular formula prediction; peaks in black are noise or cannot be explained. c) Representative extracted ion chromatogram for **15** is shown. The peak correspond to the *m/z* value calculated for the compound.


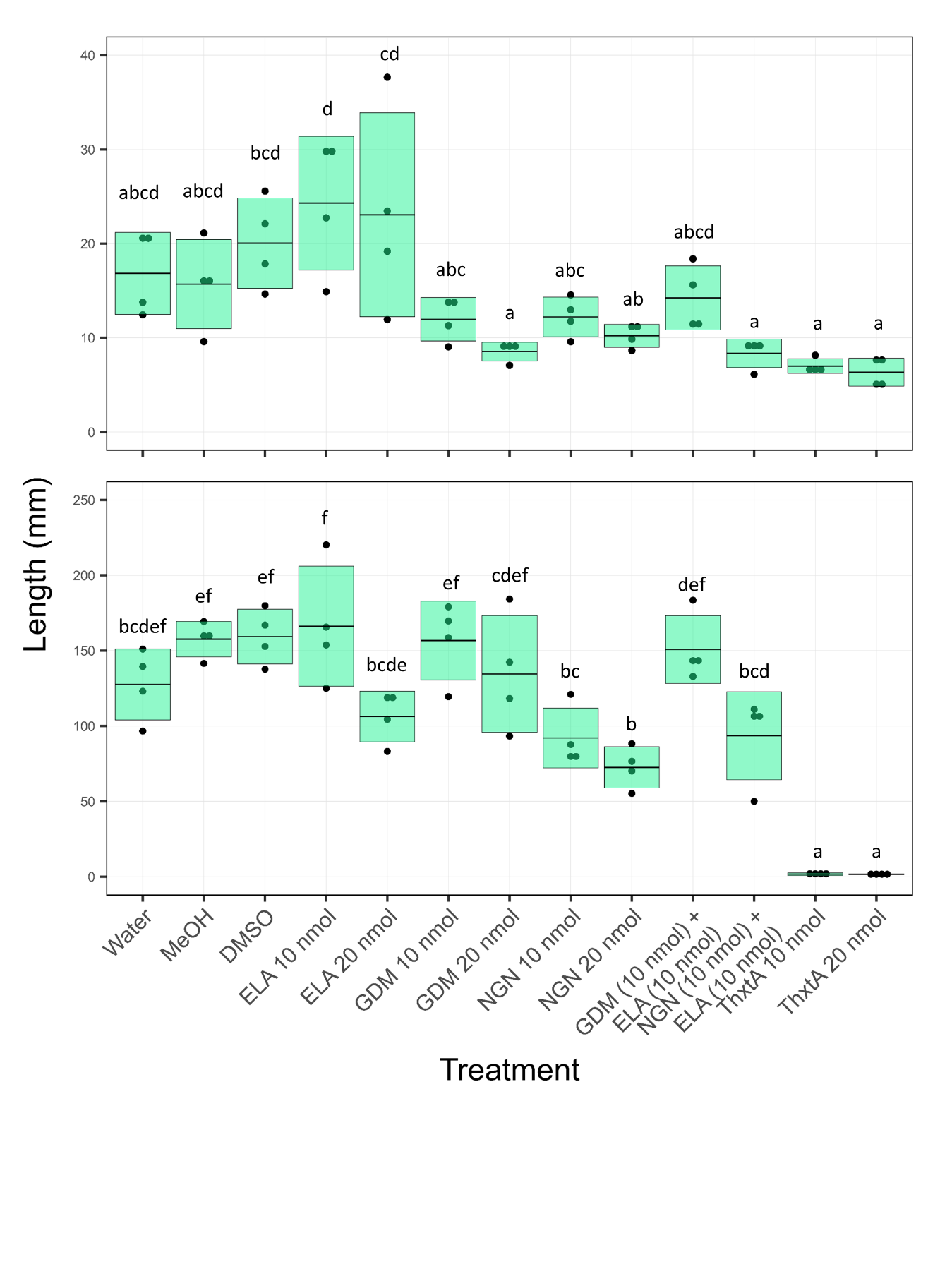


**Figure S21**. Effect of different pure compounds on radish seedlings. Elaiophylin (ELA), geldanamycin (GDM), nigericin (NGN) and thaxtomin A (ThxtA) were tested for their effects on shoot (top) and root (bottom) of radish seedlings. Seedlings were treated with 10 or 20 nmol of each compound, or a combination of 10 nmol of each compound. Each box represents the average of the measurement ± standard deviation. The data was analyzed using an ANOVA with Tukey’s test. Values with different letters are statistically different (*P* < 0.05).


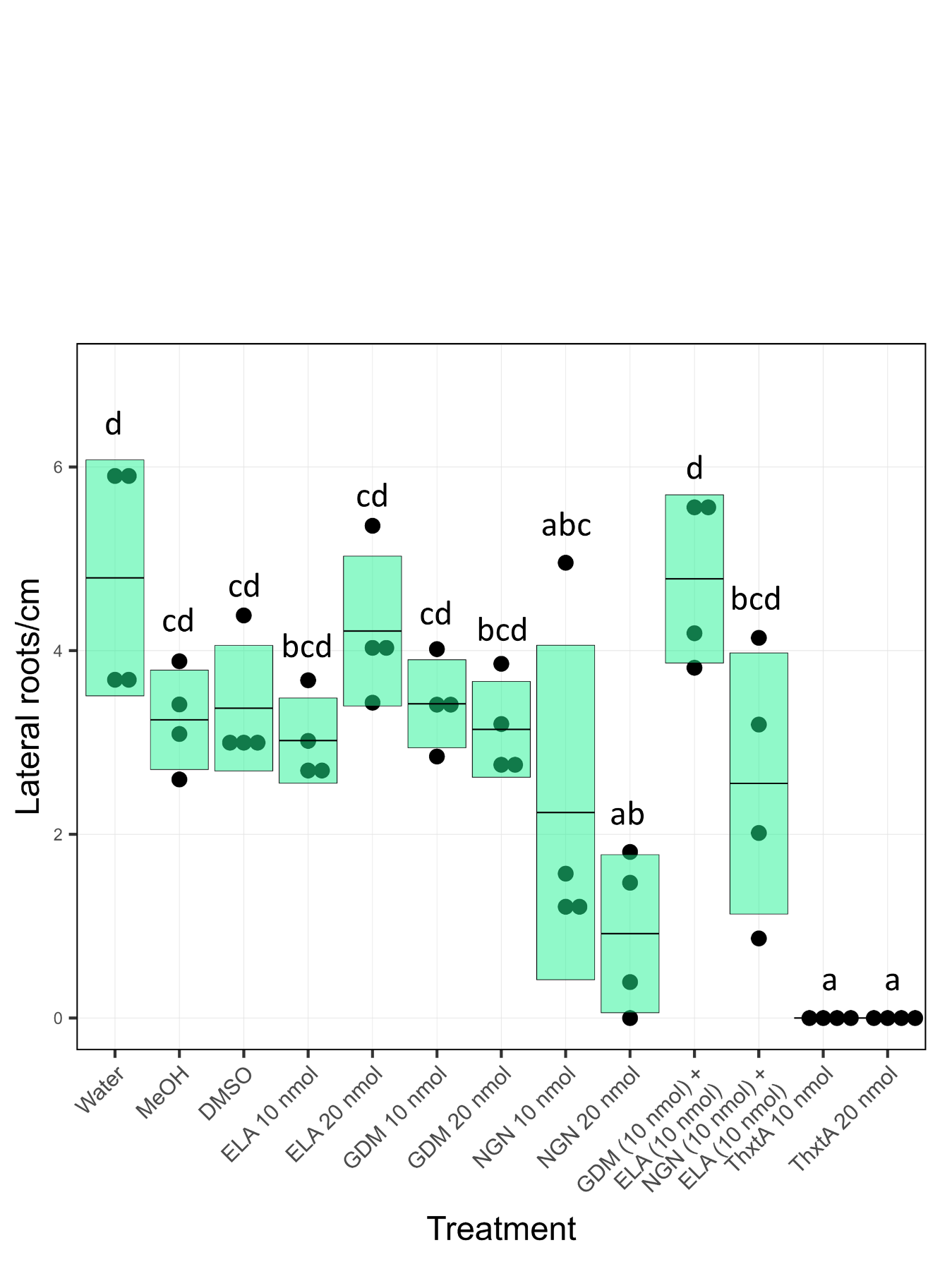


**Figure S22**. Effect of different compounds on the number of lateral roots/cm of root length in radish seedlings. Elaiophylin (ELA), geldanamycin (GDM), nigericin (NGN) and thaxtomin A (ThxtA) were tested. Each box represents the average of the measurement ± standard deviation. The data was analyzed using an ANOVA with Tukey’s test. Values with different letter are statistically different (*P* < 0.05).

**
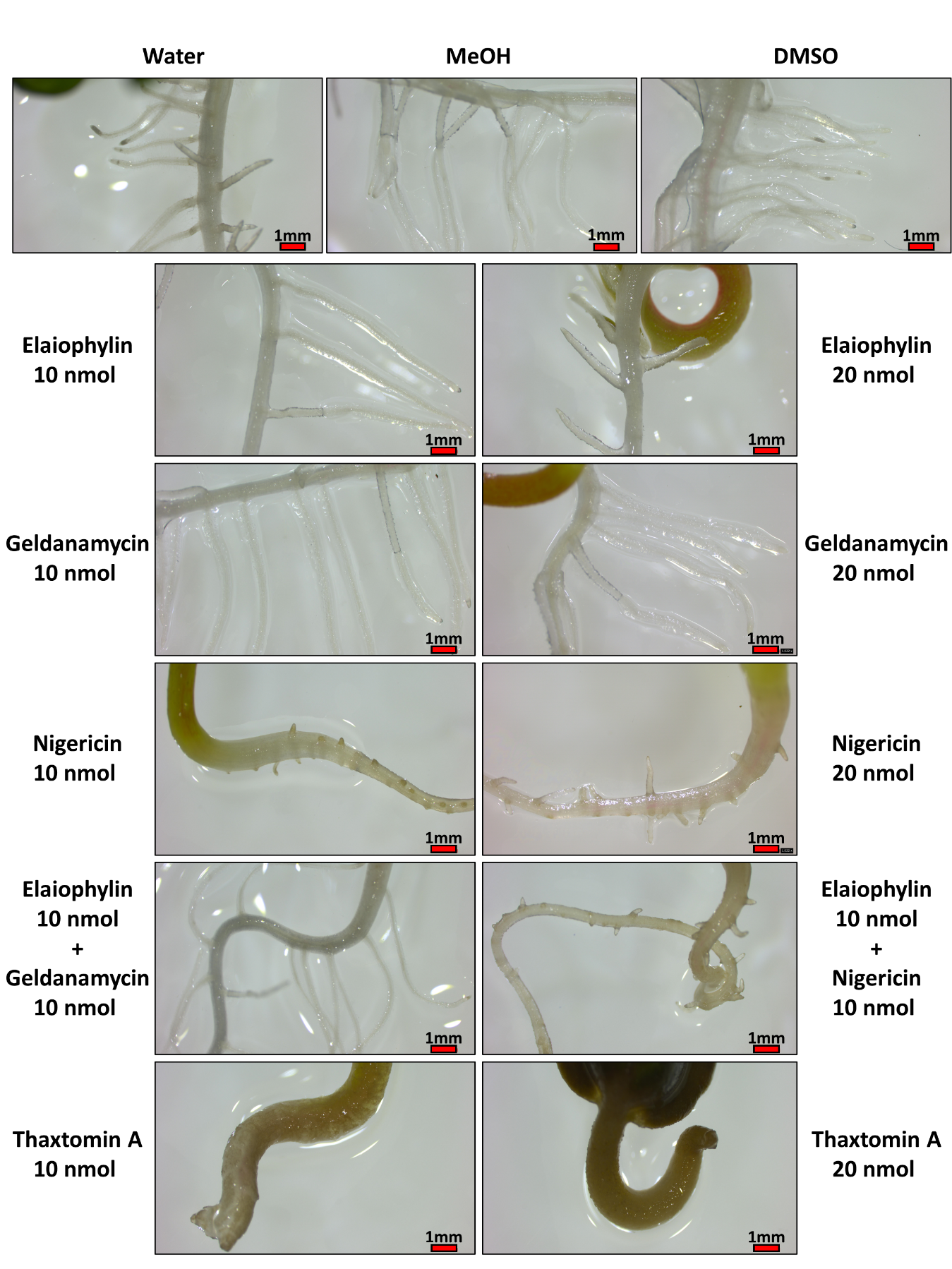
**

**Figure S23**. Effect of elaiophylin (ELA), geldanamycin (GDM), nigericin (NGN) and thaxtomin A (ThxtA) on lateral root development of radish seedlings. A 1-mm scale bar is located in the lower right corner of each photo.


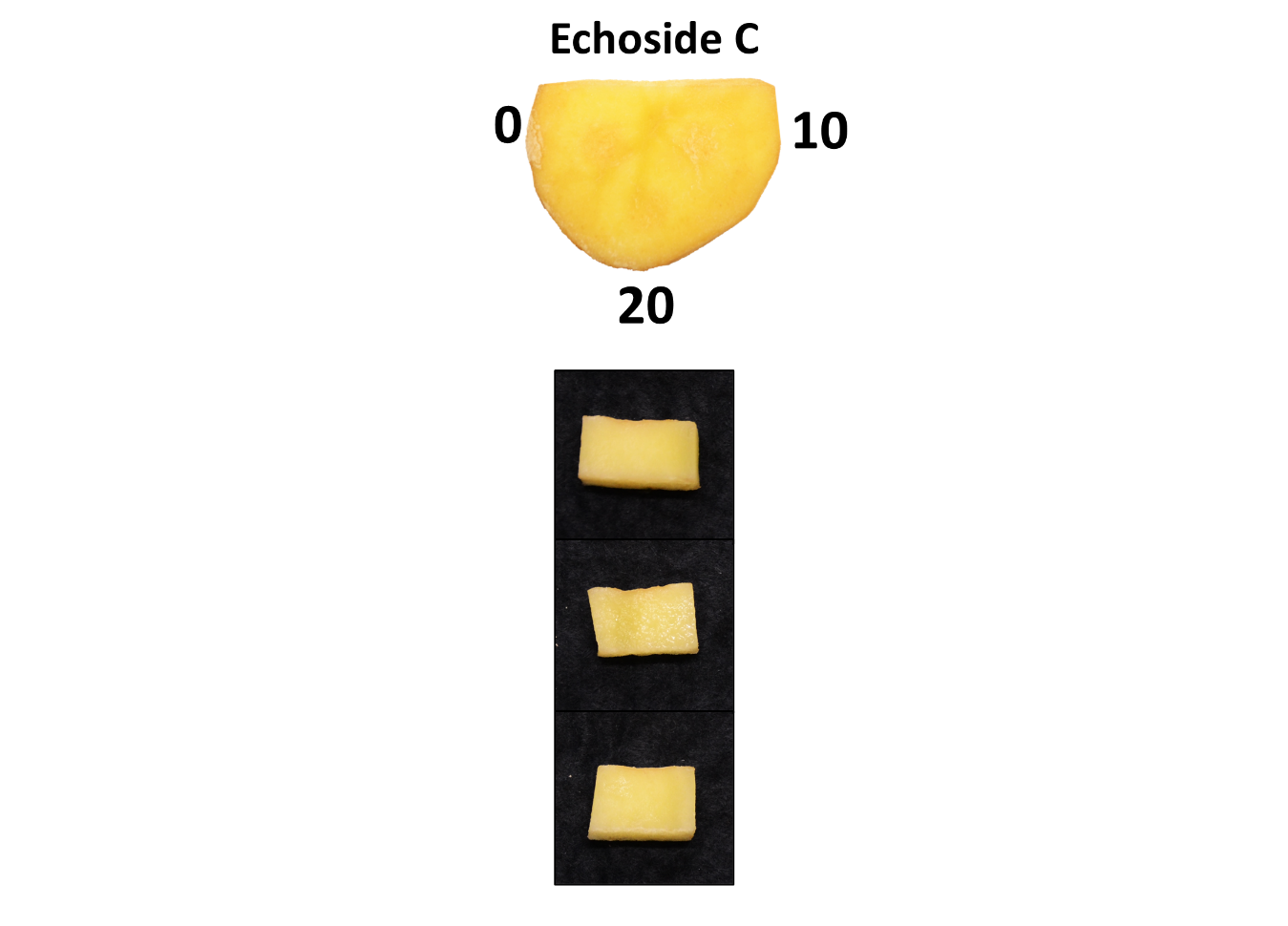


**Figure S24**. Top and side view of potato tuber slice treated with pure echoside C. The tuber slice contained disks inoculated with 0 (control), 10, and 20 nmol of the respective compound in a fixed volume of 20 μL. Three biological replicates per treatment were, with similar results obtained each time.


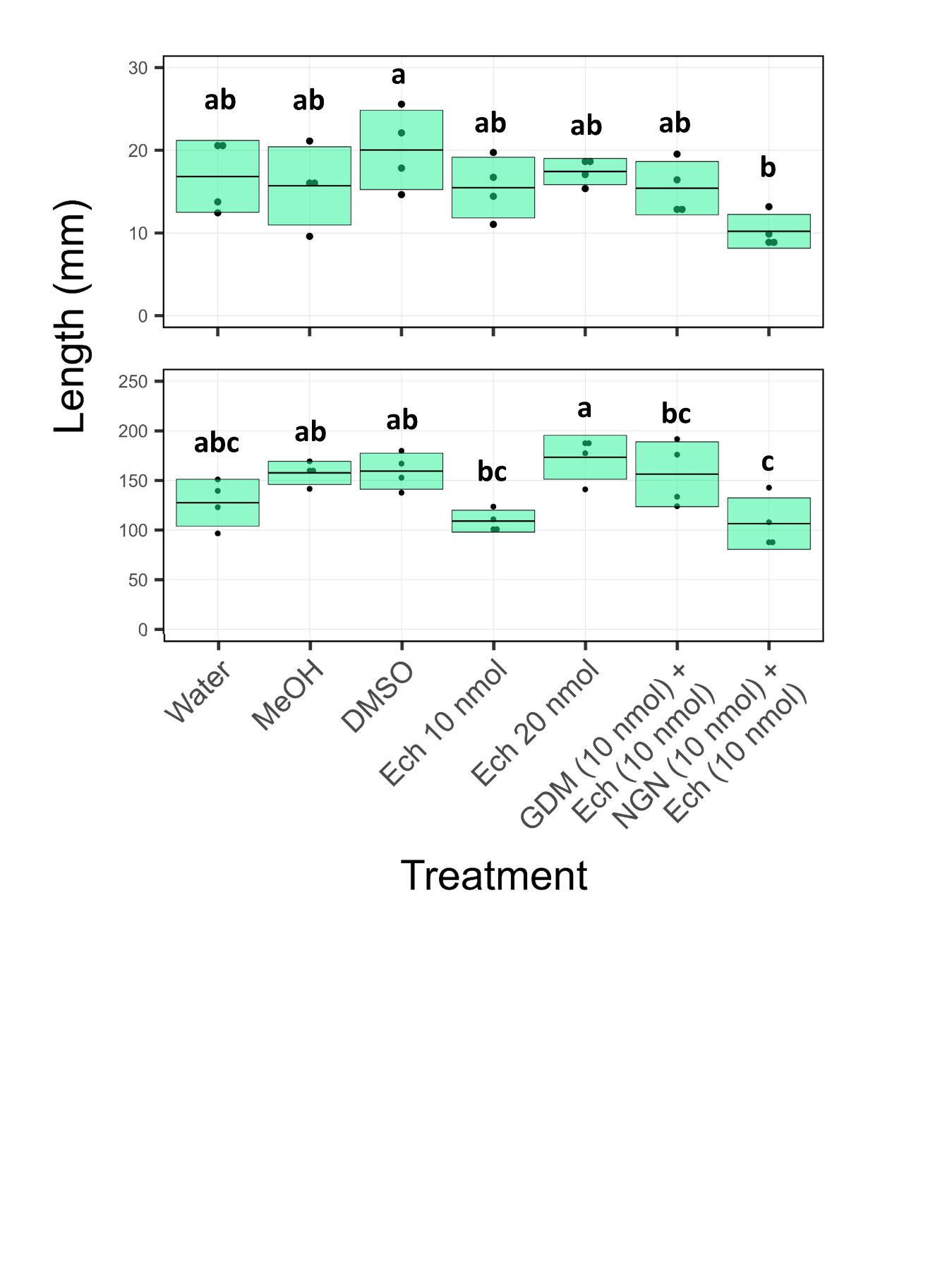


**Figure S25**. Effect of echoside C (Ech) on radish seedlings. Geldanamycin (GDM) and nigericin (NGN) were added for comparison to measure the effects on shoot (top) and root (bottom) of radish seedlings. Each box represents the average of the measurement ± standard deviation. Seedlings were treated with 10 or 20 nmol of each compound, or a combination of 10 nmol of each compound. The data was analyzed using an ANOVA with Tukey’s test. Values with different letter are statistically different (*P* < 0.05).


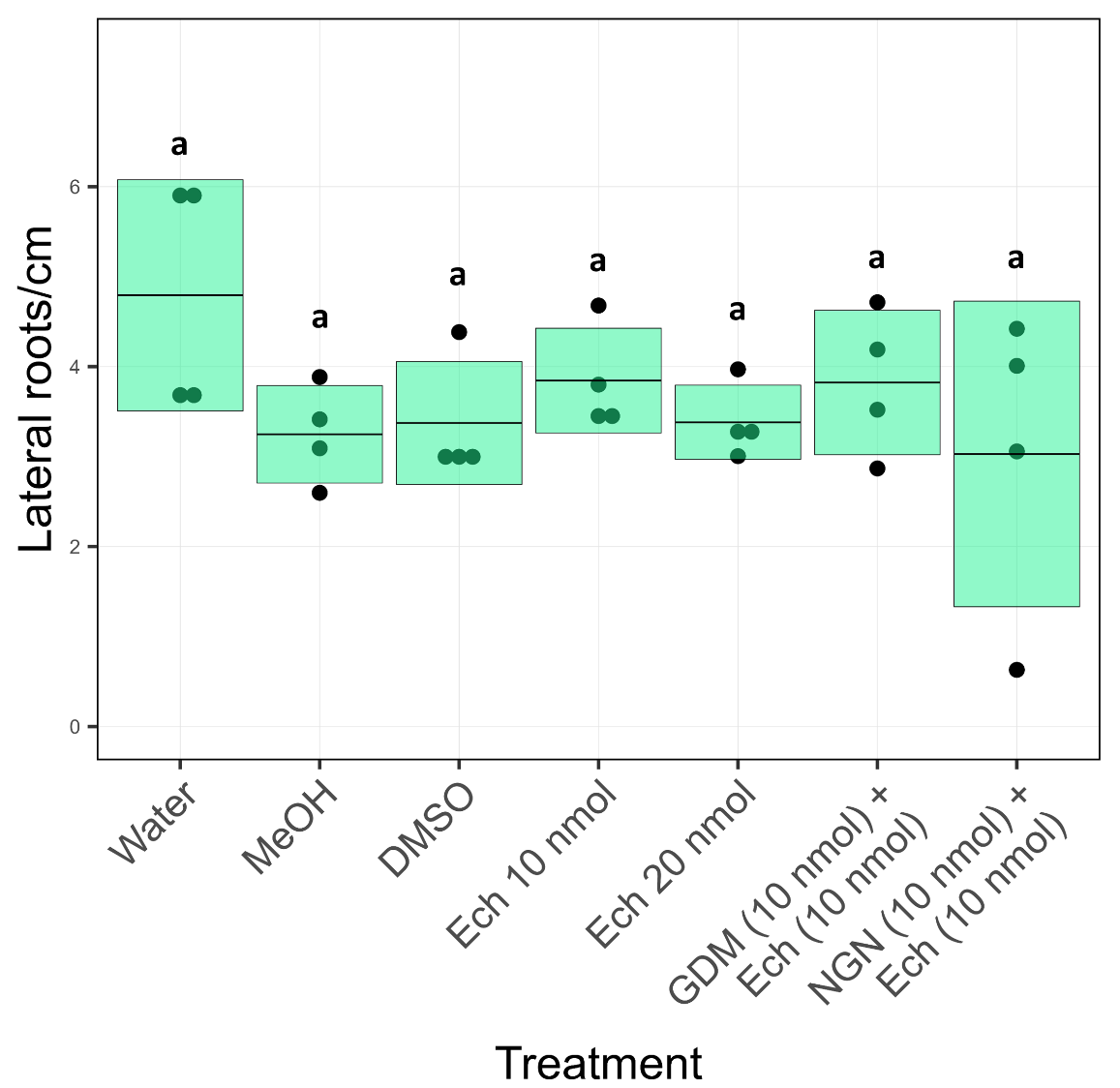


**Figure S26**. Effect of echoside C on the number of lateral roots/cm of root length in radish seedlings. Geldanamycin (GDM) and nigericin (NGN) were added to test their combined effects with echoside C. Each box represents the average of the measurement ± standard deviation. The data was analyzed using an ANOVA with Tukey’s test. Values with different letter are statistically different (*P* < 0.05).

**Table S1.** Biosynthetic gene clusters predicted in the genome of *Streptomyces* sp. 11-1-2 using antiSMASH 7.1.0.

| **Region** | **Type** | **From** | **To** | **Most similar known cluster** | | **Similarity** |
| --- | --- | --- | --- | --- | --- | --- |
| 1 | lanthipeptide-class-i | 244,013 | 268,088 | mycotrienin I | NRP+Polyketide | 7% |
| 2 | terpene | 302,342 | 328,052 | isorenieratene | Terpene | 85% |
| 3 | arylpolyene,NRPS | 457,194 | 530,146 | coprisamide C/coprisamide D | NRP | 95% |
| 4 | NRPS-like | 571,618 | 614,356 | echoside A/echoside B/echoside C/echoside D/echoside E | NRP | 11% |
| 5 | PKS-like,terpene | 658,010 | 705,819 | rustmicin | Polyketide:Iterative type I polyketide | 20% |
| 6 | terpene | 823,668 | 845,431 | brasilicardin A | Terpene+Saccharide | 38% |
| 7 | NRPS | 883,865 | 962,521 | cyclofaulknamycin | Polyketide | 8% |
| 8 | RiPP-like,T1PKS,hglE-KS | 972,458 | 1,097,361 | hexacosalactone A | Other | 79% |
| 9 | NRPS-like | 1,160,016 | 1,201,773 | meilingmycin | Polyketide | 2% |
| 10 | 2dos | 1,235,619 | 1,274,181 | hygrocin A/hygrocin B | Polyketide | 67% |
| 11 | T1PKS,NRPS | 1,277,031 | 1,393,632 | meridamycin | NRP+Polyketide | 65% |
| 12 | NAPAA | 1,540,700 | 1,574,656 | paulomycin | Other | 7% |
| 13 | T1PKS | 1,574,871 | 1,706,658 | nigericin | Polyketide:Modular type I polyketide | 100% |
| 14 | T1PKS | 1,709,024 | 1,755,410 | salinomycin | Polyketide:Modular type I polyketide | 8% |
| 15 | T1PKS,hglE-KS | 2,076,324 | 2,128,172 | hexacosalactone A | Other | 11% |
| 16 | 2dos,T1PKS,NRPS-like | 2,317,281 | 2,402,983 | naphthomycin A | Polyketide | 50% |
| 17 | NRPS,nucleoside | 2,435,414 | 2,481,506 | toyocamycin | Other | 30% |
| 18 | NRPS | 2,540,682 | 2,596,630 | glycinocin A | NRP | 11% |
| 19 | terpene | 2,943,280 | 2,964,338 | aurachin C/aurachin D/aurachin SS | Polyketide+Terpene | 20% |
| 20 | T1PKS,NI-siderophore | 2,991,226 | 3,054,812 | peucechelin | NRP | 20% |
| 21 | ectoine | 3,210,416 | 3,220,820 | ectoine | Other | 100% |
| 22 | terpene | 3,717,652 | 3,738,905 |  |  |  |
| 23 | lanthipeptide-class-i | 3,920,296 | 3,944,452 |  |  |  |
| 24 | T1PKS | 4,304,277 | 4,485,379 | desulfoclethramycin/clethramycin | Other | 61% |
| 25 | RiPP-like | 4,627,469 | 4,639,844 | granaticin | Polyketide:Type II polyketide | 10% |
| 26 | HR-T2PKS | 4,651,807 | 4,698,707 | o-dialkylbenzene 1/o-dialkylbenzene 2 | Polyketide+NRP | 58% |
| 27 | NRPS | 4,752,947 | 4,797,098 | ochronotic pigment | Other | 50% |
| 28 | indole | 5,011,258 | 5,032,403 | 5-isoprenylindole-3-carboxylate β-D-glycosyl ester | Other | 61% |
| 29 | terpene | 5,594,597 | 5,616,900 | geosmin | Terpene | 100% |
| 30 | hydrogen-cyanide | 5,958,693 | 5,971,785 | aborycin | RiPP | 14% |
| 31 | NI-siderophore | 6,749,335 | 6,779,122 | legonoxamine A/desferrioxamine B/legonoxamine B | Other | 100% |
| 32 | NRPS-like | 7,294,292 | 7,337,141 | echoside A/echoside B/echoside C/echoside D/echoside E | NRP | 100% |
| 33 | NI-siderophore | 7,912,967 | 7,942,880 | kinamycin | Polyketide | 13% |
| 34 | arylpolyene,NRPS,aminocoumarin | 7,997,568 | 8,071,451 | cinnapeptin | NRP | 82% |
| 35 | RiPP-like | 8,166,050 | 8,177,396 |  |  |  |
| 36 | T1PKS | 8,238,864 | 8,295,800 | notonesomycin A | Other | 18% |
| 37 | T2PKS | 8,421,342 | 8,493,857 | spore pigment | Polyketide | 83% |
| 38 | terpene | 8,867,788 | 8,894,236 | hopene | Terpene | 76% |
| 39 | lanthipeptide-class-i | 9,096,140 | 9,120,593 | steffimycin D | Polyketide:Type II polyketide+Saccharide:Hybrid/tailoring saccharide | 16% |
| 40 | terpene | 9,209,688 | 9,231,304 | lasalocid | Polyketide | 3% |
| 41 | T1PKS | 9,358,009 | 9,406,255 |  |  |  |
| 42 | NRPS,transAT-PKS,NRPS-like | 9,419,819 | 9,496,151 | meilingmycin | Polyketide | 9% |
| 43 | butyrolactone | 9,654,449 | 9,665,381 |  |  |  |
| 44 | hserlactone | 9,675,690 | 9,696,448 | heronamide A/heronamide B/heronamide C/heronamide D/heronamide E/heronamide F | NRP+Polyketide | 8% |
| 45 | redox-cofactor | 9,815,666 | 9,837,784 | lankacidin C | NRP+Polyketide | 13% |
| 46 | T1PKS | 9,874,400 | 9,947,755 | efomycin K/efomycin L | Polyketide | 100% |
| 47 | T1PKS,NRPS-like | 10,159,028 | 10,241,572 | geldanamycin | Polyketide | 100% |
| 48 | terpene | 10,383,333 | 10,404,487 | pristinol | Terpene | 100% |
| 49 | terpene | 10,508,385 | 10,529,722 | ebelactone | Polyketide | 11% |
| 50 | T1PKS | 10,695,155 | 10,842,742 | niphimycins C-E | Polyketide | 87% |
| 51 | NAPAA | 10,939,213 | 10,973,346 | γ-poly-L-2,4-diaminobutyric acid | NRP | 25% |
| 52 | NRP-metallophore,NRPS,T1PKS | 11,085,502 | 11,205,821 | coelichelin | NRP | 90% |
| 53 | betalactone | 11,367,403 | 11,392,789 |  |  |  |

**Table S2.** Parameters used for pre-processing the metabolomics data files in MZmine.

| **Parameter** | | **IIMN_MeOH and IIMN_EtOAc** |
| --- | --- | --- |
| **Job ID in GNPS** | | **ID=255add5c6af645ccbff3c757d9c09f22 ID=b23b95e28e9347c3aad383fde03f9682** |
| Mass detection MS1 |  |  |
|  | Scans | 1 |
|  | Mass detector | 15000 |
|  | Mass list name | masses |
| Mass detection MS2 |  |  |
|  | Scans | 2 |
|  | Mass detector | 1000 |
|  | Mass list name | masses |
| Chromatogram builder - ADAP |  |  |
|  | Scans | 4 |
|  | Mass list | masses |
|  | Group intensity threshold | 500000 |
|  | Min height | 100000 |
|  | m/z tolerance | 0.01; 10 ppm |
|  | Suffix | chromatograms |
| Chromatogram deconvolution |  |  |
|  | Suffix | deconvoluted |
|  | Algorithm | Local Minimum Search: Chromatographic threshold 10%; Search minimum in RT range (min) 0.2 ; Minimum relative height 10%; Minimum absolute height 500000.0; Min ratio of peak top/edge 1.0; Peak duration range (min) 0.0-3.0 |
|  | m/z center calculation | Median |
|  | m/z range for MS2 scan pairing (Da) | 0.02 |
|  | RT range for MS2 scan pairing (min) | 0.2 |
| Isotopic peaks grouper |  |  |
|  | Suffix | deisotoped |
|  | m/z tolerance | 0.01 ; 10 ppm |
|  | Retention time tolerance | 0.5 |
|  | Monotonic shape | Checked |
|  | Maximum charge | 4 |
|  | Representative isotope | Most intense |
| Join Aligner |  |  |
|  | Peak list name | Aligned peak lists |
|  | m/z tolerance | 0.01; 10 ppm |
|  | Weight for m/z | 75 |
|  | Retention time tolerance | 0.2 |
|  | Weight for RT | 25 |
|  | Require same charge state | No |
| Peak finder (multithreaded) |  |  |
|  | Suffix | gap-filled |
|  | Intensity Tolerance | 5% |
|  | m/z tolerance | 0.01; 5 ppm |
|  | Retention time tolerance | 0.2 |
| Peak filter |  |  |
|  | Suffix | filtered |
|  | Duration (min) | 0-3 |
|  | # data points | 3-10000 |
| Duplicate Peak filter |  |  |
|  | Suffix | dup |
|  | Filter mode | New average |
|  | m/z tolerance | 0.001 or 5 ppm |
|  | RT tolerance | 0.03 |
| Peak list rows filter |  |  |
|  | Suffix | filtered |
|  | Minimum peaks in a row | 1 |
|  | Minimum peaks in an isotope pattern | 1 |
|  | Peak duration range | - |
|  | Keep or remove rows | Keep all that match criteria |
|  | Keep only peaks with MS2 scan | Checked |
|  | Reset the peak number ID | Checked |
| Metacorrelate |  |  |
|  | RT tolerance | 0.15 |
|  | Min height | 100000 |
|  | Noise level | 10000 |
|  | Correlation grouping | Checked; min data points 5, Min data points on edge 2; measure PEARSON; min feature shape correlation 85% |
|  | Feature height correlation | Checked; min data points 2, measure PEARSON; min correlation 70% |
| Ion identity networking |  |  |
|  | m/z tolerance | 0.001; 10 ppm |
|  | Check | ONE FEATURE |
|  | Min height | 100000 |
|  | Annotation refinement | No |
|  | Ion identity library | Positive; max charge 2; max mol/cluster 2 |
|  |  | Adducts: [M+H]+; [M+Na]+; [M+NH4]+; [M+2H]2+; [M+H+Na]2+ |
|  |  |  |
|  |  |  |
|  |  | Modifications: [M-H2O]; [M-2H2O] |
|  |  |  |
|  |  |  |
|  |  |  |
|  | Ion identity library | Negative; max charge 2; max mol/cluster 2 |
|  |  | Adducts: [M-H]-; [M+Cl]- |
|  |  |  |
|  |  |  |
|  |  | Modifications: [M-H2O]; [M-2H2O] |
|  |  |  |
|  |  |  |
|  |  |  |
| Add Ion identities to network | m/z tolerance | 0.001; 10 ppm |
|  | Min height | 100000 |
|  | Ion identity library | Adducts: [M+H]+; [M+Na]+; [M+K]+; [M+NH4]+; [M+2H]2+; [M+Ca]2+; [M+Fe]2+; [M+H+Na]2+; [M+H+NH4]2+; [M-H+2Na]; [M+Ca-H]+; [M+Fe-H]+ |
|  |  |  |
|  |  |  |
|  |  |  |
| Add Ion identities to network | m/z tolerance | 0.001; 10 ppm |
|  | Min height | 100000 |
|  | Ion identity library | Adducts: [M-H]-; [M-2H+Na]-; [M+Cl]-; [M+FA]- |
|  |  |  |
|  |  |  |
|  |  | Modifications: [M-H2O]; [M-2H2O]; [M-NH3] |
| Ion identity network refinement | Minimum size | 3 |
|  | Delete small networks | Checked |
|  | Delete smaller networks | 5 |
|  | Delete networks without monomer | Checked |
| Check all ion identities by MS/MS | Mass list | masses |
|  | m/z tolerance | 0.02 or 15 ppm |
|  | Min height | 1000 |
|  | Check for multimers | Checked |
|  | Check for neutral losses | Checked; precursor |

**Table S3.** GNPS parameters used to create the molecular networks using Ion Identity Molecular Networking.

| **Parameter** | |  |  |
| --- | --- | --- | --- |
| Basic options |  |  |  |
|  | Precursor Ion Mass Tolerance | 0.02 |  |
|  | Fragment Ion Mass Tolerance | 0.02 |  |
| Advanced Network Options |  |  |  |
|  | Min pairs Cos | 0.7 |  |
|  | Min matched fragment ions | 6 |  |
| Any other parameter not included here was used as the default value from the workflow. The spectra was then compared against the GNPS spectral libraries and only results with a cosine value equal or higher than 0.7 and at least 6 fragment ions were kept. | | |  |
|  |  |  |  |
|  |  |  |  |
|  |  |  |  |
|  |  |  |  |
|  |  |  |  |
